# Rhizosphere Ion Composition Shapes Microbial Communities and Is Associated with Plant Growth Variation in Saline–Alkali Soils

**DOI:** 10.3390/microorganisms14061333

**Published:** 2026-06-14

**Authors:** Xiang Wan, Xuezhu Yao, Shengyin Zhang, Shuncun Zhang, Qi Yin

**Affiliations:** 1 Gansu Desert Control Research Institute, Wuwei 733000, China; honeywanxiang@hotmail.com; 2 Jingtai County Agro-Tech Extension and Service Center, Baiyin 730400, China; 3 Northwest Institute of Eco-Environment and Resources, Chinese Academy of Sciences, Lanzhou 730000, China; zhangshuncun@nieer.ac.cn (S.Z.); yinqihtw@163.com (Q.Y.)

**Keywords:** saline–alkali soil, ion composition, rhizosphere microbial community, plant growth status, salt ion stress, microbial community structure

## Abstract

Soil salinization severely constrains plant growth, yet the roles of ion composition and rhizosphere microbial communities in shaping plant performance remain poorly resolved. Here, we investigated multiple crop and wild plant species in saline–alkali soils and compared rhizosphere ion composition, microbial communities, and plant growth status. Restricted plant growth was consistently associated with elevated Na^+^ and Cl^−^ concentrations, while fungal diversity was significantly higher in well-growing plants. Ion composition (particularly Na^+^, Cl^−^, SO_4_^2–^, and Mg^2+^) was strongly correlated with microbial community structure, and a set of microbial taxa, including bacterial phyla such as Deinococcota and Gemmatimonadota and fungal phyla within Ascomycota and Basidiomycota, were repeatedly associated with plant growth status across species. Notably, plant species exhibited distinct apparent, threshold-like responses, and in several cases, plant growth differences were not fully explained by salinity levels alone, suggesting that rhizosphere microbial communities may buffer salt stress. Together, our results reveal that ion composition governs plant growth not only through direct ionic stress but also via microbially mediated pathways, highlighting an ion–microbe–plant interaction framework underlying growth variation in saline–alkali soils.

## 1. Introduction

Soil salinization is a key environmental issue limiting agricultural productivity and ecosystem stability worldwide. Under the combined effects of improper irrigation, rising groundwater levels, and climate change, the area of saline–alkali soil continues to expand, severely restricting sustainable land use and crop yields [1,2]. Saline–alkali conditions affect plants through multiple mechanisms, including osmotic stress, ion toxicity, and nutrient imbalance, by altering soil physicochemical properties such as pH, electrical conductivity (EC), and ion composition. These changes also significantly influence soil microbial community structure and ecological functions [1,3].

The rhizosphere microbial community is an important regulatory factor for plant adaptation to salt–alkali stress [4]. Previous studies have shown that microorganisms can enhance plant salt tolerance by regulating ion balance, synthesizing plant hormones, promoting nutrient cycling, and alleviating oxidative stress [4,5,6]. In saline environments, microbial community structures are often restructured, with salt-tolerant groups becoming enriched while sensitive groups decline [4]. However, saline–alkali soils are not homogeneous; even under similar pH conditions, ion composition can vary significantly across regions or among plant rhizospheres [7,8].

Most previous studies have simplified saline environments into single gradients (e.g., pH or EC) [3,9], with limited attention to differences in ion composition and their effects on microbial community structure. Particularly within the same saline–alkaline soil, there are significant growth differences among different plant individuals, and their driving mechanisms are still unclear. There is evidence to suggest that ion composition not only directly affects plant growth, but may also indirectly influence plant–soil interactions by regulating microbial communities [10,11]. It is worth noting that plant responses to saline–alkali stress often exhibit threshold characteristics; that is, when soil salinity or specific ion concentrations reach a certain range, plants transition from a relatively stable growth state to a restricted state [12]. During this process, plant physiological activities and root exudation patterns may change, thereby affecting their ability to regulate the rhizosphere microbial community. Therefore, apparent salinity thresholds not only determine plant growth status but may also play a key role in plant–microbe interactions [12,13]. However, systematic research is still lacking on whether different plants exhibit heterogeneous microbial responses near apparent salinity thresholds and how this process is regulated by ion composition.

Furthermore, how ion composition regulates rhizosphere microbial assembly and leads to changes in plant growth within the same saline–alkali soil remains largely unresolved. Therefore, this study investigates the rhizosphere soils of multiple plant species in saline–alkali environments, analyzes the soil physicochemical properties, microbial community structure, and their relationship with ion composition under different plant growth conditions. The aim was to clarify the coupling mechanisms among plants, ions, and microorganisms, and to provide a theoretical basis for understanding plant growth variation in saline–alkaline environments.

## 2. Materials and Methods

### 2.1. Soil Sampling

Soil samples were collected on 18 October 2025, from a typical saline–alkali soil in Caowotan Town, Jingtai County, Gansu Province, China (37.16° N, 104.13° E). The site had been previously cultivated with various crops, including cereal crops (maize, barley, and oat), oil crops (rapeseed and sunflower), and forage crops (Suaeda and Sesbania). Several wild plants, such as reed, tamarisk, and thistle, were also naturally distributed in the area. Seven commonly cultivated crops and three wild plant species were selected. Rhizosphere soil samples were collected at a depth of 10–20 cm (Supplementary File S1). Rhizosphere soil was operationally defined as the soil tightly adhering to the root surface (approximately within 2–5 mm of the root surface) after gently shaking off loosely attached soil. For each plant species, two areas with clearly different growth conditions were selected: normal growth (NG) and restricted growth (RG). The NG and RG areas of the same species were selected within the same field or adjacent soil patches to minimize large-scale environmental variation. The primary difference between groups was plant growth status. The NG group represented relatively favorable conditions for plant growth, while the RG group included smaller plants growing under near or potentially beyond the apparent salinity threshold conditions. For each plant growth condition (NG and RG), four independent rhizosphere soil samples were collected as biological replicates and processed separately for physicochemical and microbial analyses. Sequencing was performed on each replicate individually. After collection, each soil sample was immediately divided into two portions. One portion was frozen in liquid nitrogen and stored at −80 °C for microbial analysis, while the other was air-dried for physicochemical analysis.

### 2.2. Determination of Soil Physicochemical Properties

Soil pH, EC, and ion concentrations (Na^+^, K^+^, Ca^2+^, Mg^2+^, Cl^−^, SO_4_^2−^, CO_3_^2−^, and HCO_3_^−^) were measured following the methods described by Wang et al. (2024) [14] and Yan et al. (2023) [15]. Accordingly, soil pH was determined in a soil-water suspension (1:2.5, *w*/*v*). In short, soil pH was determined by the glass electrode method. A 4 g air-dried soil sample was weighed and mixed evenly with deionized water in a ratio of 1:2.5 (*w*/*v*). After standing for 30 min, a PB-10 digital pH meter (Sartorius, Göttingen, Germany) was used for measurement, and the average of is three readings was taken. Weigh 4 g of air-dried soil sample, mix soil and deionized water evenly in a ratio of 1:5 (*w*/*v*), and use DDS-307A conductivity meter (Precision and Scientific Instrument Co., Shanghai, China) to take measurements. The contents of Na^+^ and K^+^ were determined by flame photometry. The soil extract was sprayed into the flame, and the light intensity was measured at 589 nm and 766.5 nm, respectively, and quantified using a standard curve. The contents of Ca^2+^ and Mg^2+^ were determined using the ethylenediaminetetraacetic acid (EDTA) titration method. EDTA standard solution was titrated in an ammonia buffer solution with pH = 10, and the content was calculated using chromium black T indicator color change as the endpoint. The Cl^−^ content was titrated using the silver nitrate titration method in neutral or weakly alkaline solutions, with the formation of a brick red precipitate as the endpoint, and the content was calculated. The determination of SO_4_^2−^ content was carried out using the barium sulfate gravimetric method. BaCl_2_ was added to form a precipitate, which was then filtered, washed, and heated to a constant weight. The content was calculated by weighing [16]. Using an ion chromatograph (ECO IC Metrohm, Gallen, Switzerland), soluble ions CO_3_^2−^ and HCO_3_^−^ were determined according to the Chinese geological industry standard DZ/T 0064.49-93 [17].

### 2.3. DNA Extraction and Sequencing

According to the manufacturer’s instructions, use the E.Z.N.A.^TM^ MagBind Soil DNA Kit (Omega Bio-tek, Norcross, GA, USA; M5635-02) was used to extract genomic DNA from the soil samples. Subsequently, Qubit 4.0 (Thermo Fisher Scientific, Waltham, MA, USA) was used to measure the concentration of the DNA and evaluate its quality. Use 2× HieffR Robust PCR Master Mix (Yeasen Biotechnology, Shanghai, China; 10105ES03) was used to amplify the 16S rRNA V3–V4 primers. The forward primer was CCTACGGGNGGCWGCAG, and the reverse primer was GACTACHVGGGTATCTAATCC. The fungal amplification region was ITS2,: CTTGGTCATTTAGAGGAAGTAA as the forward primer and GCTGCGTTCTTCATCGATGC as the reverse primer. The PCR amplification system was as follows: genomic DNA 20 ng, Vn F (10 μmol/L) 1 μL, Vn R (10 μmol/L) 1 μL, 2× Hieff^®^ Robust PCR Master Mix (Yeasen Biotechnology, Shanghai, China) (total 30 µL). PCR reaction conditions were as follows: 95 °C for 3 min; 95 °C for 30 s, 45 °C for 30 s, 72 °C for 30 s, 5 cycles; 95 °C for 30 s, 55 °C for 30 s, 72 °C for 30 s, 20 cycles; and 72 °C for 5 min. The PCR products were purified using Hieff NGS^TM^ DNA Selection Beads (Yeasen Biotechnology, Shanghai, China) and Qubit^®^ 4.0Green double-stranded DNA assay for quantification and library quality was controlled using a bioanalyzer (Agilent Technologies, Santa Clara, CA, USA). The amplicon library was paired-end sequenced (2 × 250) on the Illumina Miseq system (Illumina Inc., San Diego, CA, USA) [18,19].

### 2.4. Bioinformatics Analysis

Trimmomatic (v0.33; Usadel Lab, Aachen, Germany) was used to perform quality filtering on the raw sequencing reads, and Cutadapt (v1.9.1; Martin Lab, Dortmund, Germany) was used to remove primer sequences. Then USEARCH (v11.0.667; Robert C. Edgar, Petaluma, CA, USA) was used to cluster the clean reads into operational taxonomic units (OTUs) with a similarity threshold of 97%. Prior to downstream analysis, low-abundance OTUs were filtered. The RDP database was used to classify and annotate bacteria, and the UNITE database was used to classify and annotate fungi. Alpha diversity (Chao1, Shannon, and Simpson) indices were calculated to assess microbial diversity, and *t*-tests and analysis of variance (ANOVA) were used to evaluate inter group differences [18].

### 2.5. Statistical Analysis

Data are presented as mean ± standard error (S.E., *n* = 4). One-way ANOVA followed by Duncan’s multiple range test was used for statistical comparisons. Differences were considered significant at *p* < 0.05 and highly significant at *p* < 0.01. All analyses were conducted using IBM SPSS Statistics 22 (IBM Corp., Armonk, NY, USA). Figures were generated using Origin 2022 (OriginLab Corporation, Northampton, MA, USA) and processed with Adobe Photoshop CS6 (Adobe Inc., San Jose, CA, USA).

## 3. Results

### 3.1. Ion Composition Characteristics of Rhizosphere Soil

The pH of all soil samples ranged from 8.14 to 9.19, indicating that they were generally alkaline. There were small differences among plant species and growth states. In contrast, electrical conductivity (EC) varied substantially, ranging from 415.56 to 2072.55 μS/cm (Table 1), indicating significant differences in overall salinity levels. In most crops, EC values in the RG group were higher than those in the NG group (Table 1). There were significant differences in the composition and concentration of major salt ions between samples. Na^+^, Cl^−^, and SO_4_^2−^ showed wide concentration ranges (Table 1). Within the same plant species, soils in the RG group generally exhibited higher Na^+^ and Cl^−^ contents. Among divalent cations, Ca^2+^ had a higher content in the NG group, while Mg^2+^ showed a similar but less pronounced trend. CO_3_^2−^ concentrations were relatively low overall (0.0029–0.0812 g/kg), whereas HCO_3_^−^ showed considerable variation among samples (Table 1). Overall, these results indicate that even under similar pH conditions, rhizosphere environments differ not only in total salinity (EC) but also in ion composition and ionic balance. When salinity and specific ion concentrations increase to a certain range, plant growth may transition from normal to restricted states, indicating a potential threshold-like response.

To further characterize the differences in salinity levels between growth conditions, electrical conductivity (EC) was compared between NG and RG across individual plant species. In maize, EC increased from 964.83 μS/cm (NG) to 1720.52 μS/cm (RG). Similarly, rapeseed showed an increase from 1338.27 to 2036.22 μS/cm, barley from 1197.93 to 1689.94 μS/cm, and oat from 749.89 to 1039.88 μS/cm. In sesbania, EC increased slightly from 1908.78 to 2053.84 μS/cm, while in thistle, EC increased from 1641.83 to 2018.78 μS/cm. In contrast, some plant species exhibited different patterns. In suaeda, EC remained relatively high under both NG (2005.15 μS/cm) and RG (1892.04 μS/cm) conditions. Tamarisk showed a decrease in EC from 802.52 to 415.56 μS/cm, and sunflower also showed a decrease from 1061.69 to 672.66 μS/cm between NG and RG conditions.

### 3.2. Rhizosphere Microbial Diversity

#### 3.2.1. Alpha Diversity of Bacterial and Fungal Communities

**Figure 1:**
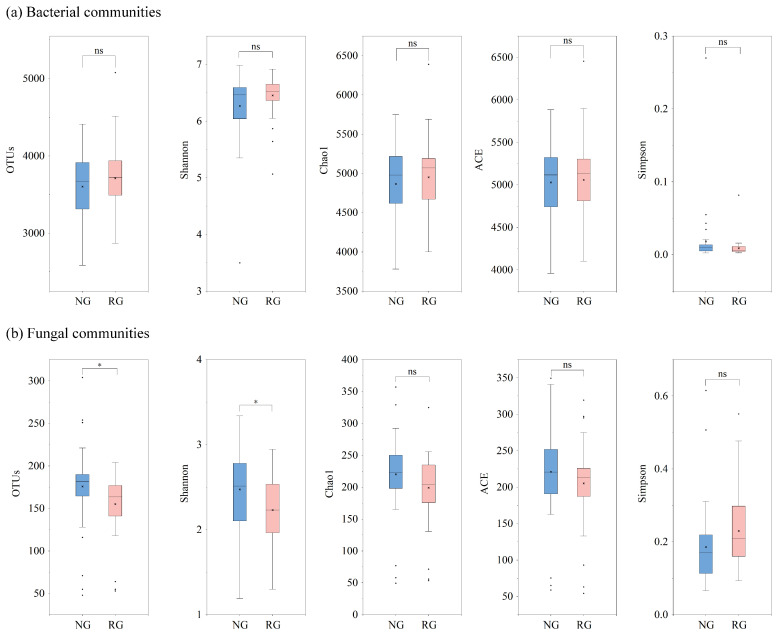
Alpha diversity indices of microbial communities in the rhizosphere soils of normal growth (NG) and restricted growth (RG) plants in saline–alkali environments. (**a**) Bacterial communities. (**b**) Fungal communities. Asterisks indicate significant correlations (* *p* < 0.05, ns *p* > 0.05).

Based on 16S rRNA and ITS sequencing data, the alpha diversity of bacterial and fungal communities in rhizosphere soils under different plant growth conditions was analyzed (Figure 1). Good’s coverage values were high for all samples (bacteria: 0.9728 ± 0.0045; fungi: 0.9989 ± 0.0007), indicating sufficient sequencing depth and reliable representation of microbial diversity. In bacterial communities, alpha diversity indices (including OTU richness, Chao1, Shannon, and ACE) were slightly higher in the RG group than in the NG group, whereas the Simpson index showed the opposite trend (Figure 1a). However, these differences were not statistically significant (*p* > 0.05), suggesting that bacterial diversity remained relatively stable under different growth conditions. In contrast, fungal communities showed more significant differences. Compared with the RG group, the rhizosphere soils of the NG group exhibited significantly higher OTU richness and Shannon diversity (*p* < 0.05), along with lower Simpson index values, indicating increased diversity and evenness (Figure 1b). These results suggest that fungal communities are more sensitive than bacterial communities to plant growth status.

#### 3.2.2. Microbial Community Composition and Dominant Taxa

**Figure 2:**
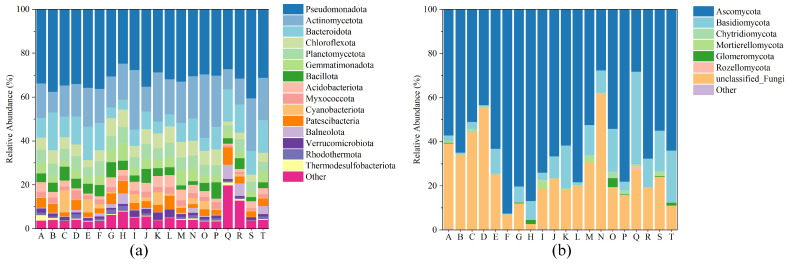
Relative abundance of bacterial (**a**) and fungal (**b**) communities in rhizosphere soils at the phylum level. Sample codes and corresponding plant species are listed in Table 1.

At the phylum level, bacterial communities were dominated by *Pseudomonadota* (24.65–40.44%), followed by *Actinomycetota* and *Bacteroidota*. *Chloroflexota* and *Planctomycetota* were also consistently present across all samples, although at relatively low abundance. Differences in the relative abundance of dominant bacterial phyla were observed between growth conditions. In general, *Actinomycetota* were more abundant in the NG group, whereas *Pseudomonadota*, *Bacteroidota*, *Chloroflexota*, and *Planctomycetota* were relatively more abundant in the RG group. However, most of these differences were not statistically significant at the phylum level (*p* > 0.05). Fungal communities were mainly composed of *Ascomycota*, *Basidiomycota*, *Chytridiomycota*, and *Mortierellomycota*, with *Ascomycota* being the dominant phylum. The relative abundance of Ascomycota was higher in the RG group, whereas Basidiomycota, Chytridiomycota, and Mortierellomycota were slightly more abundant in the NG group (Figure 2). To further characterize the dominant fungal groups within these major phyla, the fungal community composition was also examined at the genus level. The dominant fungal genera included *Alternaria*, *Botryotrichum*, *Paecilomyces*, *Naganishia*, *Scopulariopsis*, *Blumeria*, *Fusarium*, *Stemphylium*, *Vishniacozyma*, and *Coprinellus* (Supplementary Figure S1).

#### 3.2.3. Taxa Associated with Plant Growth Conditions

STAMP analysis identified a set of microbial taxa that differed significantly between growth conditions. In bacterial communities, several phyla—*Deinococcota*, *Entotheonellaeota*, *Hydrogenedentes*, and *Gemmatimonadota*—were repeatedly detected as differential taxa across multiple plant species. In fungal communities, differential taxa were mainly distributed within *Glomeromycota* and *Basidiomycota* (Supplementary File S2). To further validate these patterns, a differential abundance analysis was also performed using DESeq2. The DESeq2 results generally supported the STAMP analysis while identifying a broader range of differential taxa. In bacterial communities, phyla such as MBNT15, Chlamydiota, Deinococcota, Entotheonellaeota, and Acidobacteriota were frequently identified as differential taxa across pairwise comparisons. In fungal communities, Chytridiomycota, Mortierellomycota, Ascomycota, and Mucoromycota were among the most commonly detected differential phyla (Supplementary File S3).

### 3.3. Relationships Between Ion Composition and Microbial Communities

**Figure 3:**
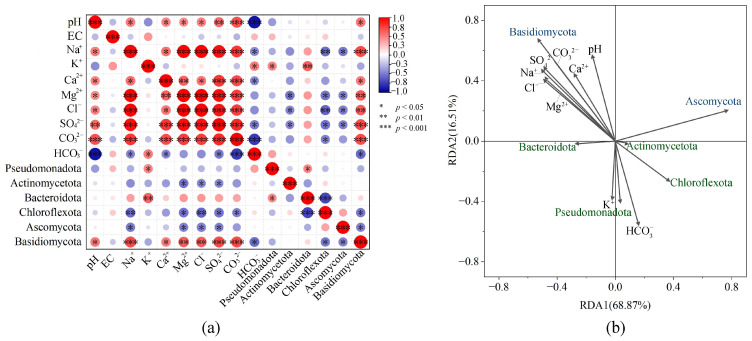
Relationships between soil ion composition and dominant microbial taxa in rhizosphere soils. (**a**) Pearson correlation analysis between major soil ions and dominant bacterial and fungal phyla. Red and blue circles indicate positive and negative correlations, respectively, and circle size represents correlation strength. Asterisks indicate significant correlations (* *p* < 0.05, ** *p* < 0.01, *** *p* < 0.001). (**b**) Redundancy analysis (RDA) showing the relationships between soil ion variables and microbial community composition. The first two axes explained 68.87% and 16.51% of the total variation, respectively.

Correlation analysis revealed significant relationships between soil ion composition and dominant microbial taxa (Figure 3a). Among the measured ions, SO_4_^2–^, Na^+^, Cl^−^ and Mg^2+^ showed significant associations with multiple dominant taxa. In bacterial communities, *Actinomycetota* were negatively correlated with SO_4_^2−^, Cl^−^ and Mg^2+^ (*p* < 0.05), while *Chloroflexota* were negatively correlated with SO_4_^2−^, Na^+^, Cl^−^, Mg^2+^, and CO_3_^2−^ (*p* < 0.05). In contrast, *Pseudomonadota* and *Bacteroidota* were positively correlated with K^+^ (*p* < 0.05). In fungal communities, *Ascomycota* showed significant negative correlations with SO_4_^2−^, Na^+^, Cl^−^, and Mg^2+^ (*p* < 0.05), whereas *Basidiomycota* were positively correlated with SO_4_^2−^, Na^+^, Cl^−^, Mg^2+^, Ca^2+^, and CO_3_^2−^, but negatively correlated with HCO_3_^−^ (*p* < 0.05). Additionally, significant positive correlations were observed among Na^+^, Ca^2+^, Mg^2+^, Cl^−^, SO_4_^2−^, and CO_3_^2−^ (*p* < 0.05), indicating coordinated variation among these ions in soil environments. Overall, these results suggest that ion composition is closely associated with changes in rhizosphere microbial community structure.

RDA showed that the first two axes explained 85.38% of the total variation in microbial community structure (RDA1: 68.87%; RDA2: 16.51%). *Basidiomycota* was positively correlated with Na^+^, Cl^−^, SO_4_^2−^, Mg^2+^, Ca^2+^, and pH, whereas *Ascomycota* and several bacterial taxa, including *Chloroflexota* and *Actinomycetota*, were distributed in the opposite direction, indicating negative correlations with these variables. *Pseudomonadota* and *Bacteroidota* were aligned with K^+^ and HCO_3_^−^ vectors (Figure 3b). The convergence of saline ion vectors suggests that these ions jointly drive variations in microbial community structure. Overall, ion composition was the main environmental factor explaining microbial community differentiation.

## 4. Discussion

### 4.1. Heterogeneity of Ion Composition in Saline–Alkali Soils

The characteristics of saline–alkaline soil are characterized not only by an increase in salinity levels, but also by significant differences in ion composition [7,20]. In this study, although soil pH remained relatively stable across samples, EC and the concentrations and relative proportions of major ions (Na^+^, Cl^−^, SO_4_^2−^, and Ca^2+^) varied considerably. This indicates that even under similar alkaline conditions, there is heterogeneity in ion composition, highlighting the inadequacy of pH alone in describing the chemical complexity of saline-alkali environments [20]. Among these ions, the Na^+^ level increased, especially when the Na^+^/Ca^2+^ ratio was imbalanced. Na^+^ can disrupt the stability of soil structure and damage plant physiological processes, while Ca^2+^ can alleviate sodium-induced stress by maintaining membrane integrity and soil aggregation. High Na^+^ and Na^+^/Ca^2+^ ratios were observed in the rhizosphere of growth-restricted plants, indicating that ion imbalance rather than absolute salinity may be a key factor limiting plant growth [21,22,23]. In addition, the ion changes most consistently associated with the restricted growth (RG) condition were characterized by the co-occurrence of increased Na^+^ and Cl^−^. This result suggests that NaCl-type salinity may play a more important role in plant growth limitation. Furthermore, there is a significant positive correlation among multiple ions (e.g., Na^+^, Cl^−^, SO_4_^2−^), which may reflect common geochemical processes such as evaporation-driven salt concentration. This co-variation implies that plants and microorganisms are exposed to complex ionic environments rather than single ion stress, which may lead to more severe ecological effects [24,25].

### 4.2. Differential Responses of Microbial Communities to Ion Environment and Plant Growth Status

The rhizosphere microbial community is an important regulatory factor for plants to adapt to salt–alkali stress [26]. This study showed distinct responses of bacterial and fungal communities to plant growth conditions. Bacterial alpha diversity did not vary significantly between groups, suggesting strong environmental filtering under saline stress that allows only salt tolerant taxa to persist. In contrast, fungal communities exhibited significantly higher diversity in the rhizosphere of well-growing plants, indicating that fungal communities are more sensitive to changes in plant physiological status and rhizosphere resources [27,28,29]. Fungi also play important roles in organic matter decomposition and nutrient cycling. Higher fungal diversity may enhance organic matter turnover, nutrient release, and soil structure, thereby creating more favorable conditions for plant growth [30,31,32]. These findings support the idea that bacteria and fungi occupy different ecological niches and respond differently to abiotic and biotic drivers [8].

In terms of community composition, dominant bacterial phyla (*Pseudomonadota*, *Actinomycetota*, and *Bacteroidota*) and fungal phyla (*Ascomycota* and *Basidiomycota*) are commonly reported in high salinity environments, reflecting their adaptability [27,33,34]. For example, *Actinomycetota*, which were more abundant in the NG group, are known for degrading complex organic matter and producing bioactive compounds that may enhance soil nutrient availability and plant health [35,36,37]. Some representative Basidiomycota genera identified in this study, such as *Naganishia*, *Vishniacozyma*, and *Filobasidium*, have also been reported in soil and rhizosphere environments under saline or other stressful conditions, suggesting that members of Basidiomycota may contribute to microbial adaptation in saline–alkali ecosystems [5,38,39]. Although overall differences in dominant taxa were limited, several microbial groups (e.g., *Deinococcota*, *Gemmatimonadota*, and specific fungal species) were consistently enriched in multiple plant species (Supplementary File S2). This suggests that they are more closely associated with specific rhizosphere conditions than with plant species per se. These taxa may play important roles in biogeochemical cycling under saline conditions [40].

Soil chemical properties are key environmental drivers of microbial community structure. In this study, SO_4_^2−^, Na^+^, and Cl^−^ were significantly associated with multiple microbial taxa. This indicates that ion composition and its balance are critical factors shaping microbial communities in saline ecosystems [6,8,28]. Different microbial taxa showed differential responses to ion composition, likely reflecting differences in physiological adaptation and ecological strategies [41]. However, it should be emphasized that correlation does not imply causation. These relationships may also be influenced by other covarying factors such as EC, nutrient availability, or plant species effects [11,42].

### 4.3. Potential Mechanisms of Plant Microbial Interactions Driven by Ionic Composition

**Figure 4:**
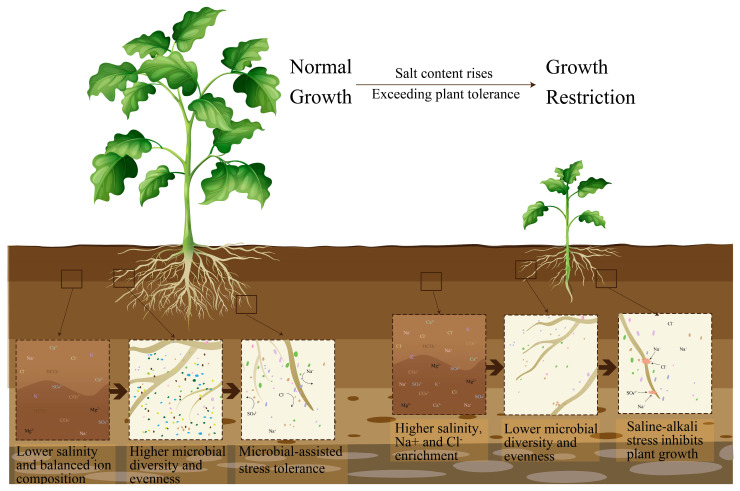
Proposed framework of ion-driven plant–microbe interactions associated with plant growth variation in saline–alkali soils.

The results suggest that ion composition may indirectly influence plant growth through a pathway involving environmental filtering, microbial community restructuring, and changes in rhizosphere ecological functions. When the concentration of certain salt ions is high, the rhizosphere environment may be more conducive to the enrichment of salt-tolerant microbial communities, and changes in the structure of these microbial communities may affect soil nutrient cycling, phytohormone production, and stress resistance, thereby indirectly affecting plant growth [26,43,44]. Changes in root exudation patterns may also contribute to the observed plant–microbe interactions. Root exudates, which include carbohydrates, organic acids, amino acids, and other metabolites, serve as an important carbon source for rhizosphere microorganisms. Previous studies have shown that salt stress can alter root exudation composition and quantity, thereby influencing microbial community assembly [45,46]. However, root exudates were not measured in this study, and their role remains to be further investigated. Based on these findings, we propose a potential mechanism: different ion compositions in saline–alkali soils may affect the growth status of plants by regulating the composition and ecological functions of rhizosphere microbial communities, leading to significant differences in plant growth within the same saline–alkali soils (Figure 4). These results also suggest that ion composition is not only a key chemical factor affecting plant growth but may also participate in plant–soil interactions through microbially mediated pathways. Therefore, regulating soil ion composition and optimizing rhizosphere microbial communities may represent effective strategies to alleviate salt stress and improve crop performance. However, this hypothesis requires further validation through experimental studies, such as microbial functional analysis or ion manipulation experiments.

### 4.4. Plant-Dependent Responses to Apparent Salinity Threshold and Associated Microbial Variation

The comparison of EC values across plant species indicates that salinity is generally consistent with plant growth status. For most cultivated crops, higher EC values were associated with restricted growth (RG), suggesting that increased salinity is an important factor limiting plant performance. This pattern is also consistent with the observed increases in Na^+^ and Cl^−^ concentrations, further supporting the role of salinity in driving plant growth differences [20]. However, several exceptions were observed. In species such as sunflower, Tamarisk, and Suaeda, EC values under RG conditions were lower than or similar to those under NG conditions, despite clear differences in plant growth status. Notably, in these cases, most measured ions (including Na^+^, Cl^−^, and SO_4_^2−^) showed consistent trends with EC, indicating that both total salinity and ion concentrations were not consistently aligned with plant growth limitation. These results suggest that salinity, although important, is not the sole factor controlling plant growth in saline–alkali soils. In these exceptional cases, differences in rhizosphere microbial communities may provide an additional explanation. Our results showed that, under some conditions, higher microbial diversity and evenness, along with the enrichment of salt-tolerant or plant growth-promoting taxa, were associated with improved plant performance [26,27]. These microbial communities may contribute to buffering salt stress through mechanisms such as nutrient cycling, ion regulation, or stress alleviation. Therefore, plant growth responses in saline–alkali soils are likely regulated by the combined effects of salinity and biological factors, rather than by salinity alone [47,48]. This highlights the importance of considering plant–microbe interactions when evaluating plant responses to salinity stress.

### 4.5. Limitations and Future Perspectives

Plant species identity is a major determinant of rhizosphere microbial community assembly and may introduce confounding effects in cross-species comparisons. In this study, we partially addressed this issue by comparing normal growth (NG) and restricted growth (RG) conditions within each plant species, thereby minimizing within-species variability. However, because multiple plant species were analyzed simultaneously, species-specific effects cannot be fully disentangled from growth-status-associated effects in cross-species analyses. Therefore, the observed microbial differences should be interpreted with caution when generalizing across plant species. Future studies should adopt factorial experimental designs or single-species controlled systems to better separate the effects of plant identity and salinity-driven environmental filtering on rhizosphere microbial communities. In addition, soil nutrient parameters were not measured in the present study. While our work focused on ion composition as the primary chemical driver of plant–microbe interactions in saline–alkali soils, the absence of nutrient data may limit a more comprehensive interpretation of plant performance and microbial community variation. Integrating nutrient availability with ion composition in future studies would provide a more holistic understanding of rhizosphere processes under salinity stress.

## 5. Conclusions

This study demonstrated that rhizosphere ion composition differed significantly under different plant growth conditions in saline–alkali soils. Higher Na^+^ and Cl^−^ concentrations were consistently associated with restricted plant growth, while fungal diversity was higher in well-growing plants, suggesting a potential microbial contribution to stress mitigation. Based on EC, plant species exhibited different apparent salinity thresholds, reflected in species-specific tolerance ranges and growth transition patterns, following the order: oat < maize < barley < rapeseed < sesbania < thistle, while Suaeda and reed exhibited relatively higher salinity tolerance. Overall, these findings highlight that plant growth in saline–alkali soils is jointly regulated by ion composition and rhizosphere microbial communities.

## Figures and Tables

**Table 1:** Soil physicochemical properties and ion composition of rhizosphere soils under different plant growth conditions.

	No.	pH	EC (μs/cm)	Na^+^ (g/kg)	K^+^ (g/kg)	Ca^2+^ (g/kg)	Mg^2+^ (g/kg)	Cl^−^ (g/kg)	SO_4_^2−^ (g/kg)	CO_3_^2−^ (g/kg)	HCO_3_^−^ (g/kg)
Maize (NG)	A	8.37 ± 0.85	964.83 ± 88.15	0.4442 ± 0.0846	0.1230 ± 0.0187	1.1179 ± 0.0316	0.1421 ± 0.0823	0.4300 ± 0.0025	3.0845 ± 0.4983	0.0138 ± 0.0012	0.2376 ± 0.0729
Maize (RG)	B	8.28 ± 0.91	1720.52 ± 50.85	0.8797 ± 0.0844	0.3810 ± 0.0170	1.9127 ± 0.4151	0.2845 ± 0.0625	0.4793 ± 0.0613	6.9565 ± 1.6903	0.0046 ± 0.0015	0.2782 ± 0.0512
Rapeseed (NG)	C	8.32 ± 0.18	1338.27 ± 53.02	1.0163 ± 0.0040	0.1487 ± 0.0072	0.8276 ± 0.3645	0.2726 ± 0.0614	1.2595 ± 0.0430	3.1175 ± 0.5534	0.0129 ± 0.0010	0.2028 ± 0.0915
Rapeseed (RG)	D	8.14 ± 0.57	2036.22 ± 88.75	1.8999 ± 0.0155	0.5921 ± 0.0377	1.1276 ± 0.2235	0.4884 ± 0.1564	2.0780 ± 0.0466	3.8545 ± 0.9193	0.0151 ± 0.0017	0.3014 ± 0.0388
Suaeda (NG)	E	8.56 ± 0.75	2005.15 ± 17.37	1.7575 ± 0.0404	0.4047 ± 0.0775	1.0846 ± 0.1059	0.4306 ± 0.0855	1.9335 ± 0.2855	4.7353 ± 0.5359	0.0233 ± 0.0013	0.2434 ± 0.0698
Suaeda (RG)	F	8.57 ± 0.43	1892.04 ± 76.25	1.5728 ± 0.0999	0.3980 ± 0.0047	1.0753 ± 0.2603	0.3765 ± 0.0831	1.7545 ± 0.5485	4.4675 ± 0.7155	0.0161 ± 0.0017	0.1710 ± 0.0085
Sesbania (NG)	G	8.45 ± 0.93	1908.78 ± 37.15	0.7850 ± 0.0176	0.0657 ± 0.0033	2.4665 ± 0.6068	0.3598 ± 0.0083	0.6185 ± 0.0374	4.9185 ± 0.5870	0.0219 ± 0.0072	0.1652 ± 0.0116
Sesbania (RG)	H	8.80 ± 0.29	2053.84 ± 59.95	1.8028 ± 0.0193	0.0649 ± 0.0024	2.3394 ± 1.0425	0.4004 ± 0.0439	1.3750 ± 0.0866	7.0735 ± 2.1586	0.0164 ± 0.0059	0.1797 ± 0.0039
Barley (NG)	I	8.24 ± 0.67	1197.93 ± 14.51	0.3518 ± 0.0899	0.0345 ± 0.0081	1.3741 ± 0.0438	0.2090 ± 0.0973	0.4489 ± 0.0518	3.9845 ± 0.5963	0.0054 ± 0.0020	0.2666 ± 0.0574
Barley (RG)	J	8.67 ± 0.29	1689.94 ± 42.73	0.8011 ± 0.0704	0.0853 ± 0.0049	1.9775 ± 0.1205	0.3554 ± 0.0433	0.6210 ± 0.0455	5.2465 ± 0.2354	0.0178 ± 0.0054	0.1681 ± 0.0101
Oat (NG)	K	8.48 ± 0.24	749.89 ± 57.75	0.2048 ± 0.0621	0.0173 ± 0.0050	0.6649 ± 0.2705	0.0985 ± 0.0256	0.1826 ± 0.0156	2.5845 ± 0.9641	0.0169 ± 0.0056	0.1768 ± 0.0045
Oat (RG)	L	8.71 ± 0.82	1039.88 ± 25.82	0.2373 ± 0.0334	0.0231 ± 0.0061	1.4659 ± 0.6404	0.1575 ± 0.0793	0.2213 ± 0.0315	3.8395 ± 0.6758	0.0206 ± 0.0055	0.1420 ± 0.0240
Reed (NG)	M	8.83 ± 0.52	2061.85 ± 82.25	2.5351 ± 0.0051	0.1063 ± 0.0039	2.1741 ± 0.9947	0.5027 ± 0.0584	1.8610 ± 0.4785	9.3498 ± 2.9298	0.0235 ± 0.0078	0.1217 ± 0.0349
Reed (RG)	N	8.84 ± 0.13	2072.55 ± 54.61	2.5796 ± 0.0306	0.1094 ± 0.0043	2.2406 ± 0.6081	0.4975 ± 0.0916	2.3720 ± 0.2252	8.7355 ± 1.7522	0.0276 ± 0.0091	0.1159 ± 0.0038
Sunflower (NG)	O	8.66 ± 0.61	1061.69 ± 93.75	0.7248 ± 0.0157	0.0727 ± 0.0039	1.0021 ± 0.2965	0.1727 ± 0.0427	0.6114 ± 0.0825	3.5158 ± 0.5665	0.0060 ± 0.0018	0.1681 ± 0.0101
Sunflower (RG)	P	8.96 ± 0.48	672.66 ± 87.92	0.6954 ± 0.0322	0.0681 ± 0.0013	0.4344 ± 0.0775	0.1008 ± 0.0709	0.4293 ± 0.0255	1.9345 ± 0.5211	0.0175 ± 0.0058	0.1130 ± 0.0395
Tamarisk (NG)	Q	9.07 ± 0.61	802.52 ± 73.45	14.0093 ± 0.4113	0.1302 ± 0.0041	3.4460 ± 1.0720	1.6599 ± 0.6815	9.9905 ± 0.9349	22.7090 ± 6.4821	0.0812 ± 0.0081	0.0608 ± 0.0672
Tamarisk (RG)	R	9.19 ± 0.97	415.56 ± 36.80	4.2031 ± 0.0863	0.0881 ± 0.0082	3.6282 ± 1.0215	0.6917 ± 0.0277	4.1980 ± 0.8845	12.3538 ± 2.1554	0.0583 ± 0.0021	0.0289 ± 0.0845
Thistle (NG)	S	8.47 ± 0.08	1641.83 ± 57.35	0.3008 ± 0.0649	0.0849 ± 0.0014	3.1491 ± 1.1285	0.1690 ± 0.0471	0.1337 ± 0.0253	6.6995 ± 1.7758	0.0166 ± 0.0055	0.2318 ± 0.0076
Thistle (RG)	T	8.91 ± 0.29	2018.78 ± 67.06	1.6294 ± 0.0176	0.0601 ± 0.0038	2.3184 ± 0.6535	0.2934 ± 0.0753	0.5860 ± 0.0754	6.1705 ± 2.4527	0.0561 ± 0.0019	0.0289 ± 0.0604

## Data Availability

The original contributions presented in this study are included in the article/Supplementary Material. Further inquiries can be directed to the corresponding authors.

## Supplementary Materials

The following supporting information can be downloaded at: https://www.mdpi.com/article/10.3390/microorganisms14061333/s1, Supplementary File S1: Representative plant samples collected from saline–alkali soils in Jingtai County, Gansu Province, China. Different plant species under normal growth (NG) and restricted growth (RG) conditions are shown; Supplementary File S2: STAMP differential analysis results. Supplementary File S3: DESeq2 differential analysis results. Supplementary Figure S1: Relative abundance of fungal communities in rhizosphere soils at the genus level. Sample codes and corresponding plant species are listed in Table 1.

## References

[1] Haj-Amor Z., Araya T., Kim D.G., Bouri S., Lee J., Ghiloufi W., Yang Y., Kang H., Jhariya M.K., Banerjee A. (2022). Soil salinity and its associated effects on soil microorganisms, greenhouse gas emissions, crop yield, biodiversity and desertification: A review. Sci. Total Environ..

[2] Ondrasek G., Rengel Z. (2021). Environmental salinization processes: Detection, implications & solutions. Sci. Total Environ..

[3] Mohanavelu A., Naganna S.R., Al-Ansari N. (2021). Irrigation Induced Salinity and Sodicity Hazards on Soil and Groundwater: An Overview of Its Causes, Impacts and Mitigation Strategies. Agriculture.

[4] Fan W., Xiao Y., Dong J., Xing J., Tang F., Shi F. (2023). Variety-driven rhizosphere microbiome bestows differential salt tolerance to alfalfa for coping with salinity stress. Front. Plant Sci..

[5] Liu Y.P., Xun W.B., Chen L., Xu Z.H., Zhang N., Feng H.C., Zhang Q., Zhang R.F. (2022). Rhizosphere microbes enhance plant salt tolerance: Toward crop production in saline soil. Comput. Struct. Biotechnol. J..

[6] Xia F., Hao H., Qi Y., Bai H., Li H., Shi Z., Shi L. (2023). Effect of Salt Stress on Microbiome Structure and Diversity in Chamomile (*Matricaria chamomilla* L.) Rhizosphere Soil. Agronomy.

[7] Wei T.J., Li G., Cui Y.R., Xie J., Gao X.A., Teng X., Zhao X.Y., Guan F.C., Liang Z.W. (2024). Variation Characteristics of Root Traits of Different Alfalfa Cultivars under Saline-Alkaline Stress and their Relationship with Soil Environmental Factors. Phyton-Int. J. Exp. Bot..

[8] Hou Y., Zeng W., Hou M., Wang Z., Luo Y., Lei G., Zhou B., Huang J. (2021). Responses of the Soil Microbial Community to Salinity Stress in Maize Fields. Biology.

[9] Shi Y., Li Y.T., Yang T., Chu H.Y. (2021). Threshold effects of soil pH on microbial co-occurrence structure in acidic and alkaline arable lands. Sci. Total Environ..

[10] Guo H., Huang Z., Li M., Hou Z. (2020). Growth, ionic homeostasis, and physiological responses of cotton under different salt and alkali stresses. Sci. Rep..

[11] Song J.J., Guan X.T., Cui H.J., Liu L., Li Y.H., Li Y., Li Y., Ma S.R. (2025). The impact of salt-tolerant plants on soil nutrients and microbial communities in soda saline-alkali lands of the Songnen plain. Front. Microbiol..

[12] He Q., Silliman B.R., Cui B.S. (2017). Incorporating thresholds into understanding salinity tolerance: A study using salt-tolerant plants in salt marshes. Ecol. Evol..

[13] Pan Y.Q., Kang P., Tan M., Hu J.P., Zhang Y.Q., Zhang J.L., Song N.P., Li X.R. (2022). Root exudates and rhizosphere soil bacterial relationships of *Nitraria tangutorum* are linked to k-strategists bacterial community under salt stress. Front. Plant Sci..

[14] Wang Y., Gong H., Zhang Z., Sun Z., Liu S., Ma C., Wang X., Liu Z. (2024). Effects of microbial communities during the cultivation of three salt-tolerant plants in saline-alkali land improvement. Front. Microbiol..

[15] Yan S.H., Zhang T.B., Zhang B.B., Zhang T.G., Cheng Y., Wang C., Luo M., Feng H., Siddique K.H.M. (2023). The higher relative concentration of K^+^ to Na^+^ in saline water improves soil hydraulic conductivity, salt-leaching efficiency and structural stability. Soil.

[16] Bao S.D. (2005). Soil Agricultural Chemical Analysis.

[17] (1993). Determination of Carbonate and Bicarbonate in Soil.

[18] Wang T., Zhang S., Zhang S., Shao M., Ding Z., Zhou Y., Su C. (2024). The Process of Soil Carbon Sequestration in Different Ecological Zones of Qingtu Lake in the Arid–Semi-Arid Region of Western China. Microorganisms.

[19] Su C., Zhang S., Zhou Y., Tan H., Zhang S., Wang T., Ding Z., Liao J. (2025). Microbial Community Response and Assembly Process of Yellow Sand Matrix in a Desert Marginal Zone Under *Morchella* Cultivation. Microorganisms.

[20] Lei S.H., Jia X.X., Zhao C.L., Shao M. (2025). A review of saline-alkali soil improvements in China: Efforts and their impacts on soil properties. Agric. Water Manag..

[21] Wei T.J., Jiang C.J., Jin Y.Y., Zhang G.H., Wang M.M., Liang Z.W. (2020). Ca^2+^/Na^+^ Ratio as a Critical Marker for Field Evaluation of Saline-Alkaline Tolerance in Alfalfa (*Medicago sativa* L.). Agronomy.

[22] Manzoor Q., Jim D.O., Sven S., Ghulam M. (2006). Vegetative bioremediation of sodic and saline-sodic soils for productivity enhancement and environment conservation. Biosaline Agriculture and Salinity Tolerance in Plants.

[23] Hadi M.R., Karimi N. (2012). The role of calcium in plants’ salt tolerance. J. Plant Nutr..

[24] Cui G., Lu Y., Zheng C., Liu Z., Sai J. (2019). Relationship between Soil Salinization and Groundwater Hydration in Yaoba Oasis, Northwest China. Water.

[25] Otlewska A., Migliore M., Dybka-Stępień K., Manfredini A., Struszczyk-Świta K., Napoli R., Białkowska A., Canfora L., Pinzari F. (2020). When Salt Meddles Between Plant, Soil, and Microorganisms. Front. Plant Sci..

[26] Yuan Z.L., Irina S.D., Labbé J., Redman R., Qin Y., Rodriguez R., Zhang C., Tuskan G.A., Lin F. (2016). Specialized Microbiome of a Halophyte and its Role in Helping Non-Host Plants to Withstand Salinity. Sci. Rep..

[27] Zhang Z.C., Feng S.C., Luo J.Q., Hao B.H., Diao F.W., Li X., Jia B.B., Wang L.X., Bao Z.H., Guo W. (2021). Evaluation of Microbial Assemblages in Various Saline-Alkaline Soils Driven by Soluble Salt Ion Components. J. Agric. Food Chem..

[28] Rath K.M., Maheshwari A., Rousk J. (2019). Linking Microbial Community Structure to Trait Distributions and Functions Using Salinity as an Environmental Filter. mBio.

[29] Wan W.J., Liu S., Li X., Xing Y.H., Chen W.L., Huang Q.Y. (2021). Bridging Rare and Abundant Bacteria with Ecosystem Multifunctionality in Salinized Agricultural Soils: From Community Diversity to Environmental Adaptation. mSystems.

[30] Wang X.L., Chi Y.K., Song S.Z. (2024). Important soil microbiota’s effects on plants and soils: A comprehensive 30-year systematic literature review. Front. Microbiol..

[31] Yu S.H., Liu P.Y., Liu Y.H., Wang Y.Q. (2025). Research Advances in Ecological Functions and Regulatory Mechanisms of the Arbuscular Mycorrhizal Fungi-Plant-Soil Interaction Network. Adv. Environ. Prot..

[32] Liu H.Q., Lu X.B., Li Z.H., Tian C.Y., Song J. (2021). The role of root-associated microbes in growth stimulation of plants under saline conditions. Land Degrad. Dev..

[33] Wang S., Sun L., Ling N., Zhu C., Chi F.Q., Li W.Q., Hao X.Y., Zhang W., Bian J.Y., Chen L. (2019). Exploring Soil Factors Determining Composition and Structure of the Bacterial Communities in Saline-Alkali Soils of Songnen Plain. Front. Microbiol..

[34] Peng M., Jia H.B., Wang Q.Y. (2017). The Effect of Land Use on Bacterial Communities in Saline-Alkali Soil. Curr. Microbiol..

[35] Zheng Y., Cao X., Zhou Y., Ma S., Wang Y., Li Z., Zhao D., Yang Y., Zhang H., Meng C. (2024). Purines enrich root-associated *Pseudomonas* and improve wild soybean growth under salt stress. Nat. Commun..

[36] Misko A.L., Germida J.J. (2002). Taxonomic and functional diversity of pseudomonads isolated from the roots of field-grown canola. FEMS Microbiol. Ecol..

[37] Yang J., Li W., Teng D., Yang X., Zhang Y., Li Y. (2022). Metagenomic Insights into Microbial Community Structure, Function, and Salt Adaptation in Saline Soils of Arid Land, China. Microorganisms.

[38] Zhu S.S., Lei Y.H., Wang C., Wei Y.M., Wang C.C., Sun Y.F. (2021). Patterns of yeast diversity distribution and its drivers in rhizosphere soil of Hami melon orchards in different regions of Xinjiang. BMC Microbiol..

[39] Liu B.S., Hu Y.H., Wang Y., Xue H.H., Li Z.H., Li M. (2022). Effects of saline-alkali stress on bacterial and fungal community diversity in Leymus chinensis rhizosphere soil. Environ. Sci. Pollut. Res..

[40] Du J., Wang Z., Hu L., Wang L., Fang J., Liu R. (2024). Comparative Genomics Reveal Distinct Environment Preference and Functional Adaptation Among Lineages of Gemmatimonadota. Microorganisms.

[41] Dong Y., Chen R., Graham E.B., Yu B., Bao Y., Li X., You X., Feng Y. (2024). Eco-evolutionary strategies for relieving carbon limitation under salt stress differ across microbial clades. Nat. Commun..

[42] Peng Y.L., Gao Z.W., Gao Y., Liu G.F., Sheng L.X., Wang D.L. (2008). Eco-physiological Characteristics of Alfalfa Seedlings in Response to Various Mixed Salt-alkaline Stresses. J. Integr. Plant Biol..

[43] Roy S., Chakraborty A.P., Chakraborty R. (2021). Understanding the potential of root microbiome influencing salt-tolerance in plants and mechanisms involved at the transcriptional and translational level. Physiol. Plant..

[44] Ruppel S., Franken P., Witzel K. (2013). Properties of the halophyte microbiome and their implications for plant salt tolerance. Funct. Plant Biol..

[45] Kumar N., Haldar S., Saikia R. (2023). Root exudation as a strategy for plants to deal with salt stress: An updated review. Environ. Exp. Bot..

[46] Upadhyay S.K., Srivastava A.K., Rajput V.D., Chauhan P.K., Bhojiya A.A., Jain D., Chaubey G., Dwivedi P., Sharma B., Minkina T. (2022). Root Exudates: Mechanistic Insight of Plant Growth Promoting Rhizobacteria for Sustainable Crop Production. Front. Microbiol..

[47] Parasar B.J., Sharma I., Agarwala N. (2021). Root exudation drives abiotic stress tolerance in plants by recruiting beneficial microbes. Appl. Soil Ecol..

[48] Feng D., Li W., Huang P., Gu M., Tang G., Ding Y., Cao G., Xu W. (2025). Mechanisms of microorganisms alleviating drought and salt stresses in plants. Microorganisms.

