# Supplementary material for: Rhizosphere Ion Composition Shapes Microbial Communities and Is Associated with Plant Growth Variation in Saline–Alkali Soils
Source: Microorganisms. 2026 Jun 14;14(6):1333. doi: 10.3390/microorganisms14061333 (PMC13305541; doi:10.3390/microorganisms14061333)
Supplement: Supplementary file 1 [file microorganisms-14-01333-s001.zip › Supplementary Figure S1.pdf]

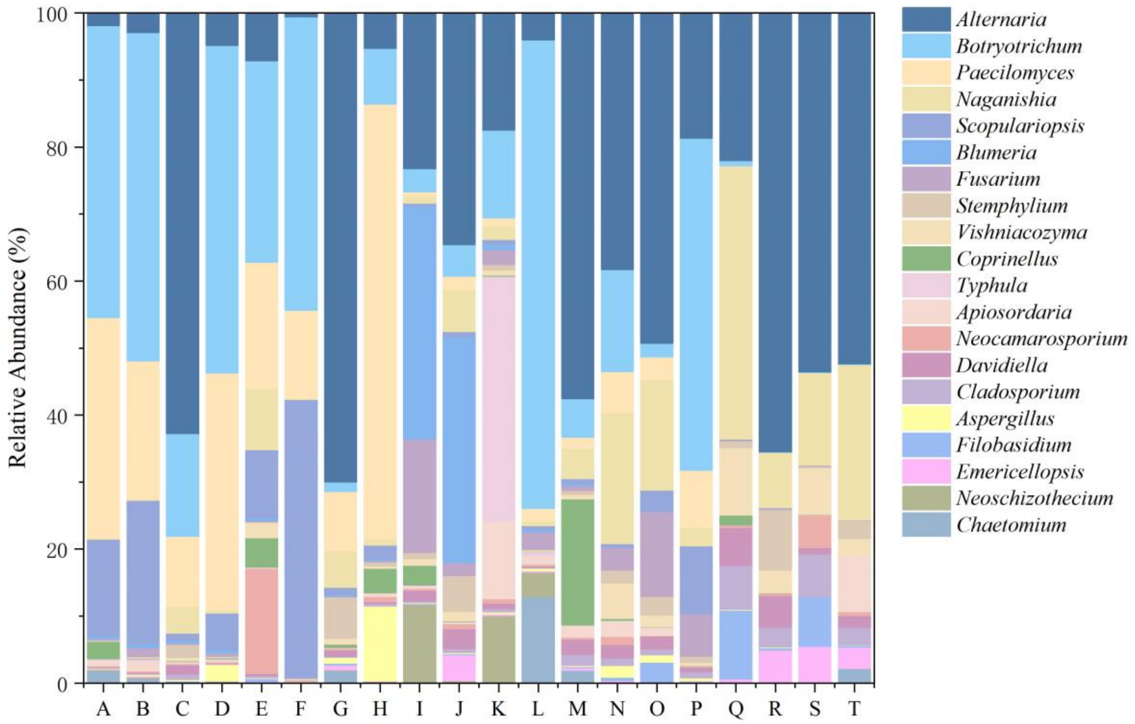


**Figure S1.** Relative abundance of fungal communities in rhizosphere soils at the genus level. Sample codes and corresponding plant species are listed in Table 1.
