# Supplementary material for: Rhizosphere Ion Composition Shapes Microbial Communities and Is Associated with Plant Growth Variation in Saline–Alkali Soils
Source: Microorganisms. 2026 Jun 14;14(6):1333. doi: 10.3390/microorganisms14061333 (PMC13305541; doi:10.3390/microorganisms14061333)

Supplementary File S1: Representative plant samples collected from saline–alkali soils in Jingtai County, Gansu Province, China. Different plant species under normal growth (NG) and restricted growth (RG) conditions are shown.

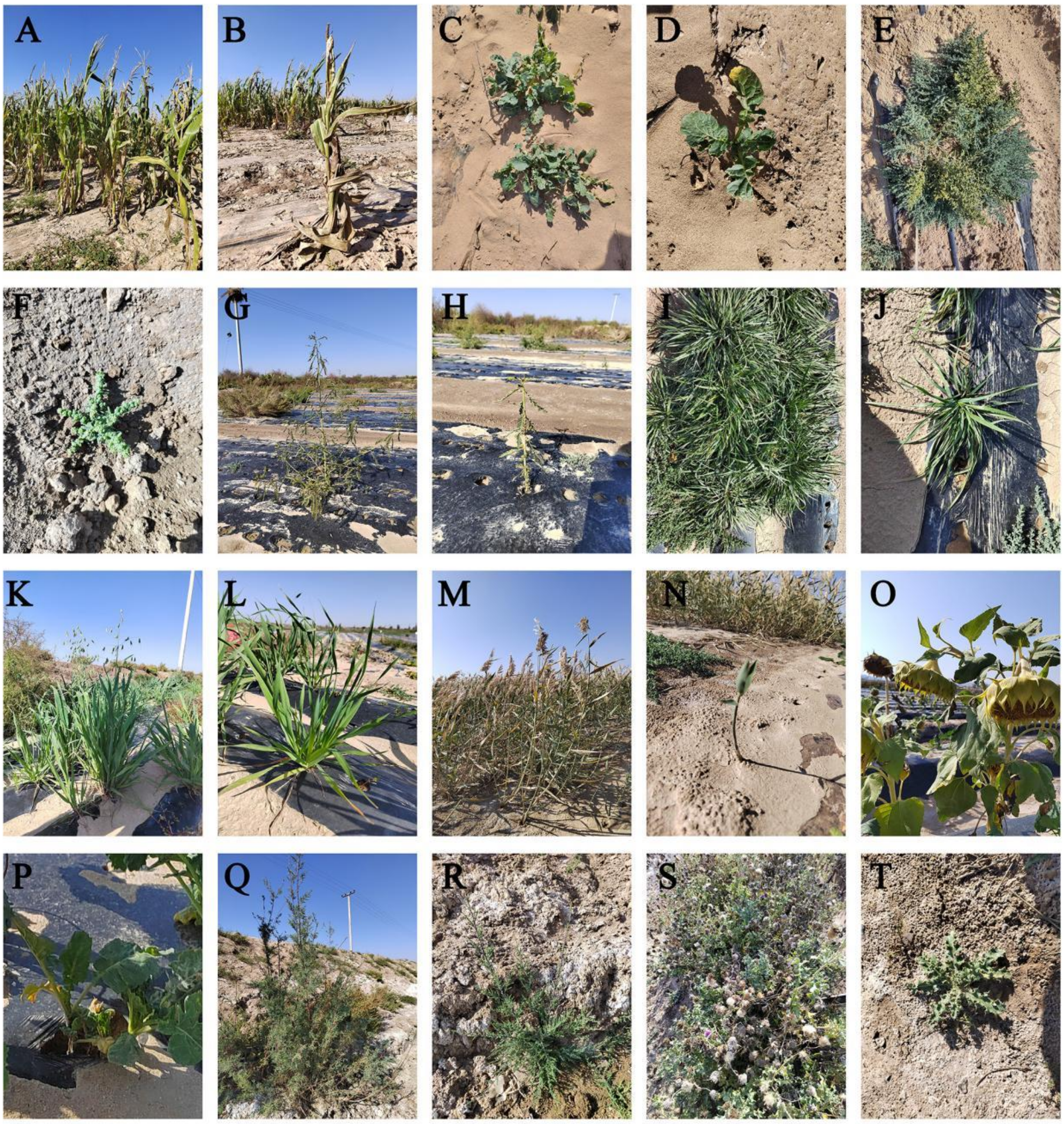

Supplement: Supplementary file 1 [file microorganisms-14-01333-s001.zip › Supplementary File S1.pdf]
