# Supplementary material for: Rhizosphere Ion Composition Shapes Microbial Communities and Is Associated with Plant Growth Variation in Saline–Alkali Soils
Source: Microorganisms. 2026 Jun 14;14(6):1333. doi: 10.3390/microorganisms14061333 (PMC13305541; doi:10.3390/microorganisms14061333)
Supplement: Supplementary file 1 [file microorganisms-14-01333-s001.zip › Supplementary File S2.pdf]

Supplementary File S2. STAMP differential analysis results

Part 1: Differential Analysis Results of Bacterial Communities

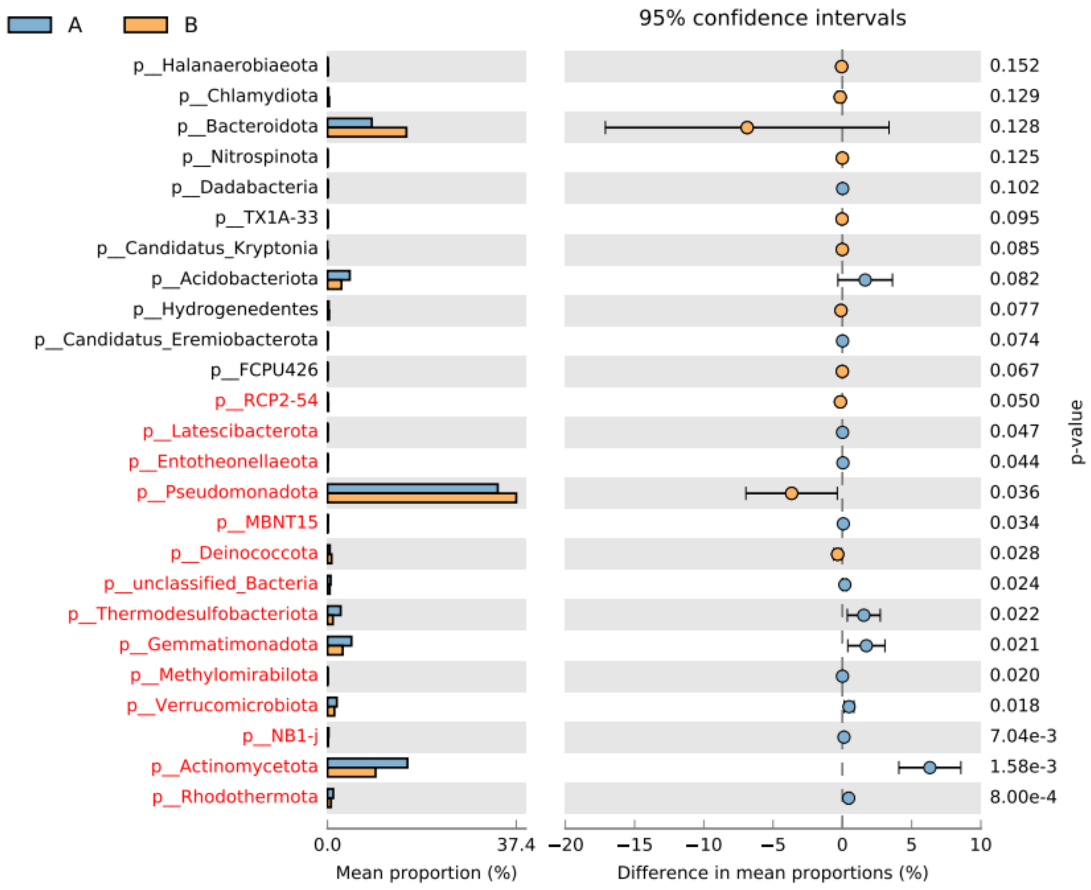

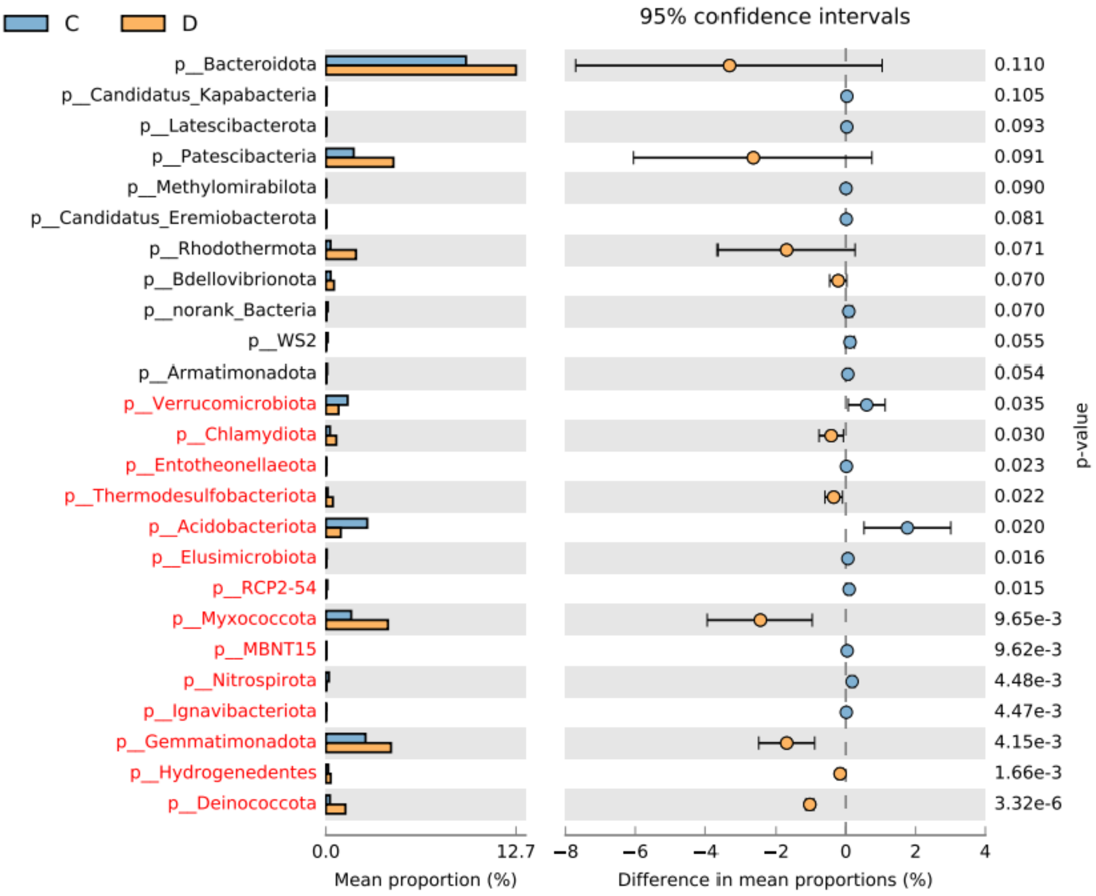
C
D
95% confidence intervals
p__Bacteroidota
p__Candidatus_Kapabacteria
p__Latescibacterota
p__Patescibacteria
p__Methylomirabilota
p__Candidatus_Eremiobacterota
p__Rhodothermota
p__Bdellovibrionota
p__norank_Bacteria
p__WS2
p__Armatimonadota
p__Verrucomicrobiota
p__Chlamydiota
p__Entotheonellaeota
p__Thermodesulfobacteriota
p__Acidobacteriota
p__Elusimicrobiota
p__RCP2-54
p__Myxococcota
p__MBNT15
p__Nitrospirota
p__Ignavibacteriota
p__Gemmatimonadota
p__Hydrogenedentes
p__Deinococcota
0.110
0.105
0.093
0.091
0.090
0.081
0.071
0.070
0.070
0.055
0.054
0.035
0.030
0.023
0.022
0.020
0.016
0.015
9.65e-3
9.62e-3
4.48e-3
4.47e-3
4.15e-3
1.66e-3
3.32e-6
p-value
0.0
12.7
Mean proportion (%)
−8
−6
−4
−2
0
2
4
Difference in mean proportions (%)

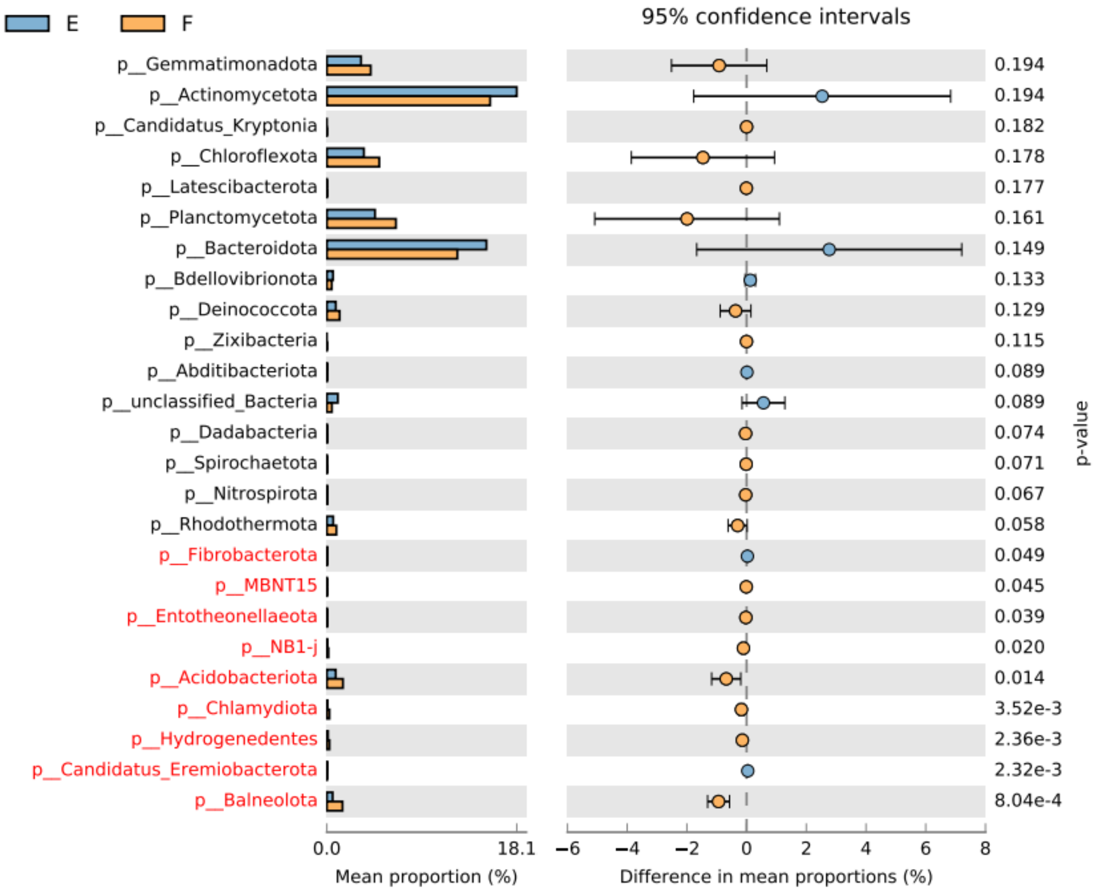
E
F
95% confidence intervals
p__Gemmatimonadota
p__Actinomycetota
p__Candidatus_Kryptonia
p__Chloroflexota
p__Latescibacterota
p__Planctomycetota
p__Bacteroidota
p__Bdellovibrionota
p__Deinococcota
p__Zixibacteria
p__Abditibacteriota
p__unclassified_Bacteria
p__Dadabacteria
p__Spirochaetota
p__Nitrospirota
p__Rhodothermota
p__Fibrobacterota
p__MBNT15
p__Entotheonellaeota
p__NB1-j
p__Acidobacteriota
p__Chlamydiota
p__Hydrogenedentes
p__Candidatus_Eremiobacterota
p__Balneolota
0.194
0.194
0.182
0.178
0.177
0.161
0.149
0.133
0.129
0.115
0.089
0.089
0.074
0.071
0.067
0.058
0.049
0.045
0.039
0.020
0.014
3.52e-3
2.36e-3
2.32e-3
8.04e-4
p-value
0.0
18.1
Mean proportion (%)
−6
−4
−2
0
2
4
6
8
Difference in mean proportions (%)

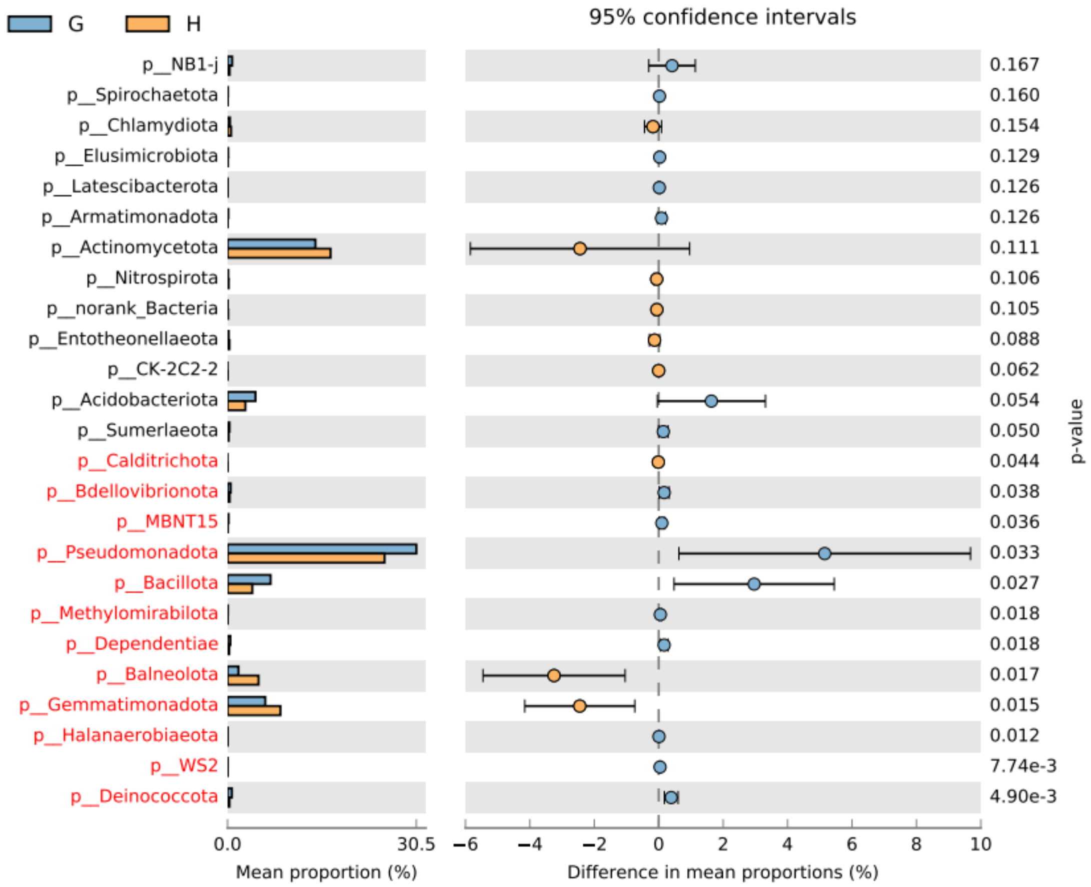
G
H
95% confidence intervals
p__NB1-j
p__Spirochaetota
p__Chlamydiota
p__Elusimicrobiota
p__Latescibacterota
p__Armatimonadota
p__Actinomycetota
p__Nitrospirota
p__norank_Bacteria
p__Entotheonellaeota
p__CK-2C2-2
p__Acidobacteriota
p__Sumerlaeota
p__Calditrichota
p__Bdellovibrionota
p__MBNT15
p__Pseudomonadota
p__Bacillota
p__Methylomirabilota
p__Dependentiae
p__Balneolota
p__Gemmatimonadota
p__Halanaerobiaeota
p__WS2
p__Deinococcota
0.167
0.160
0.154
0.129
0.126
0.126
0.111
0.106
0.105
0.088
0.062
0.054
0.050
0.044
0.038
0.036
0.033
0.027
0.018
0.018
0.017
0.015
0.012
7.74e-3
4.90e-3
p-value
0.0
30.5
Mean proportion (%)
−6
−4
−2
0
2
4
6
8
10
Difference in mean proportions (%)

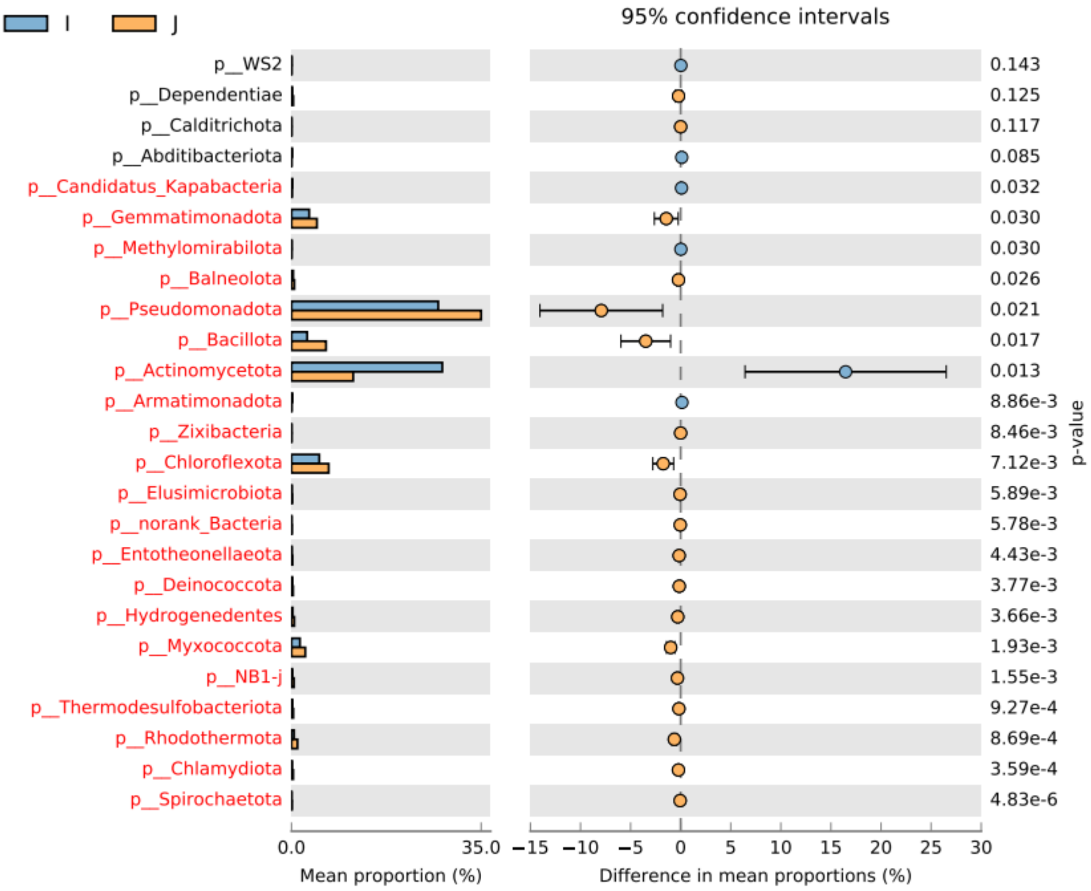
I
J
95% confidence intervals
p__WS2
p__Dependentiae
p__Calditrichota
p__Abditibacteriota
p__Candidatus_Kapabacteria
p__Gemmatimonadota
p__Methylomirabilota
p__Balneolota
p__Pseudomonadota
p__Bacillota
p__Actinomycetota
p__Armatimonadota
p__Zixibacteria
p__Chloroflexota
p__Elusimicrobiota
p__norank_Bacteria
p__Entotheonellaeota
p__Deinococcota
p__Hydrogenedentes
p__Myxococcota
p__NB1-j
p__Thermodesulfobacteriota
p__Rhodothermota
p__Chlamydiota
p__Spirochaetota
0.143
0.125
0.117
0.085
0.032
0.030
0.030
0.026
0.021
0.017
0.013
8.86e-3
8.46e-3
7.12e-3
5.89e-3
5.78e-3
4.43e-3
3.77e-3
3.66e-3
1.93e-3
1.55e-3
9.27e-4
8.69e-4
3.59e-4
4.83e-6
p-value
0.0
35.0
Mean proportion (%)
−15
−10
−5
0
5
10
15
20
25
30
Difference in mean proportions (%)

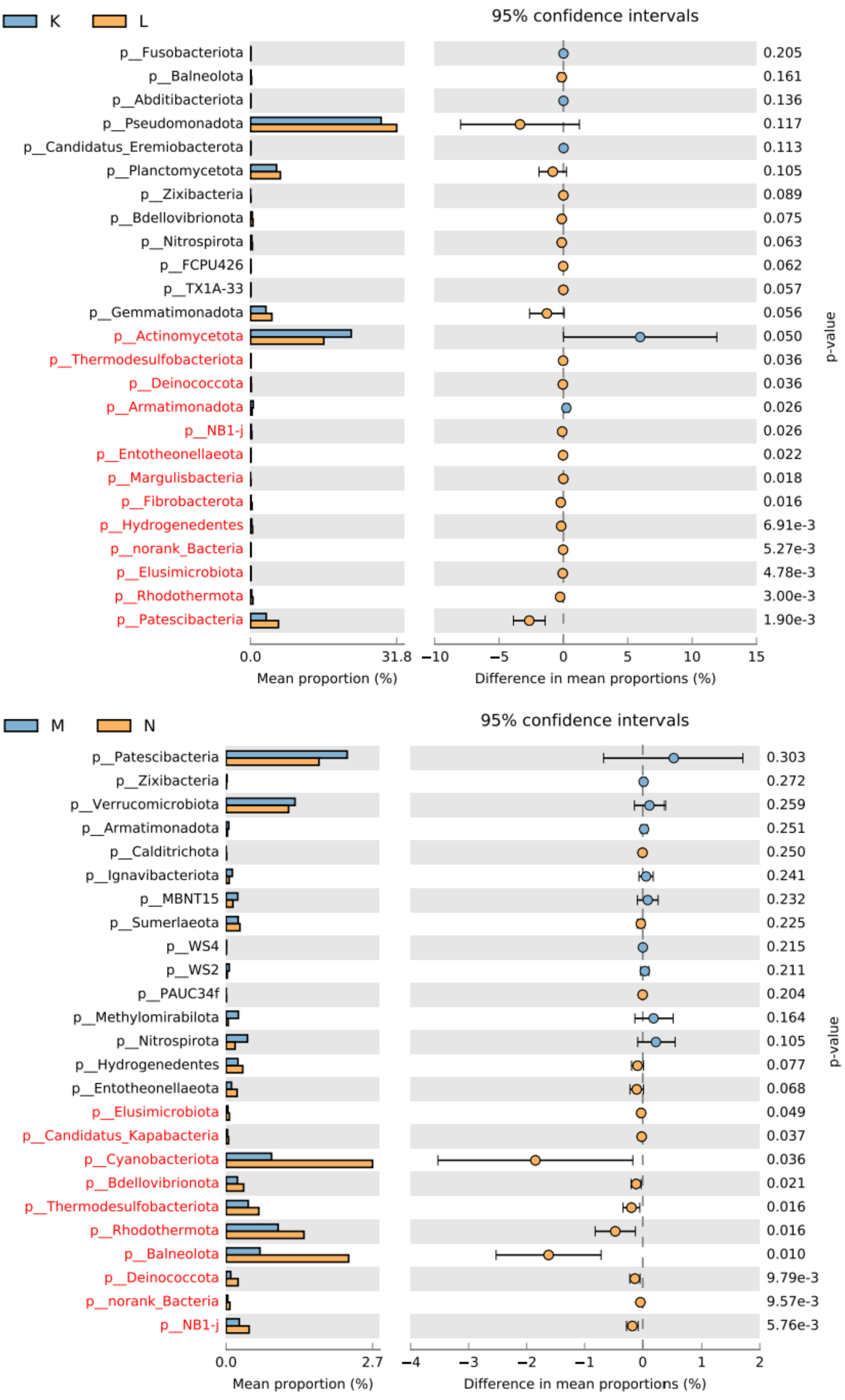

K
L
95% confidence intervals
p__Fusobacteriota 0.205
p__Balneolota 0.161
p__Abditibacteriota 0.136
p__Pseudomonadota 0.117
p__Candidatus_Eremiobacterota 0.113
p__Planctomycetota 0.105
p__Zixibacteria 0.089
p__Bdellovibrionota 0.075
p__Nitrospirota 0.063
p__FCPU426 0.062
p__TX1A-33 0.057
p__Gemmatimonadota 0.056
p__Actinomycetota 0.050
p__Thermodesulfobacteriota 0.036
p__Deinococcota 0.036
p__Armatimonadota 0.026
p__NB1-j 0.026
p__Entotheonellaeota 0.022
p__Margulisbacteria 0.018
p__Fibrobacterota 0.016
p__Hydrogenedentes 6.91e-3
p__norank_Bacteria 5.27e-3
p__Elusimicrobiota 4.78e-3
p__Rhodothermota 3.00e-3
p__Patescibacteria 1.90e-3
0.0
31.8
Mean proportion (%)
−10 −5 0 5 10 15
Difference in mean proportions (%)
p-value
M
N
95% confidence intervals
p__Patescibacteria 0.303
p__Zixibacteria 0.272
p__Verrucomicrobiota 0.259
p__Armatimonadota 0.251
p__Calditrichota 0.250
p__Ignavibacteriota 0.241
p__MBNT15 0.232
p__Sumerlaeota 0.225
p__WS4 0.215
p__WS2 0.211
p__PAUC34f 0.204
p__Methylomirabilota 0.164
p__Nitrospirota 0.105
p__Hydrogenedentes 0.077
p__Entotheonellaeota 0.068
p__Elusimicrobiota 0.049
p__Candidatus_Kapabacteria 0.037
p__Cyanobacteriota 0.036
p__Bdellovibrionota 0.021
p__Thermodesulfobacteriota 0.016
p__Rhodothermota 0.016
p__Balneolota 0.010
p__Deinococcota 9.79e-3
p__norank_Bacteria 9.57e-3
p__NB1-j 5.76e-3
0.0
2.7
Mean proportion (%)
−4 −3 −2 −1 0 1 2
Difference in mean proportions (%)
p-value

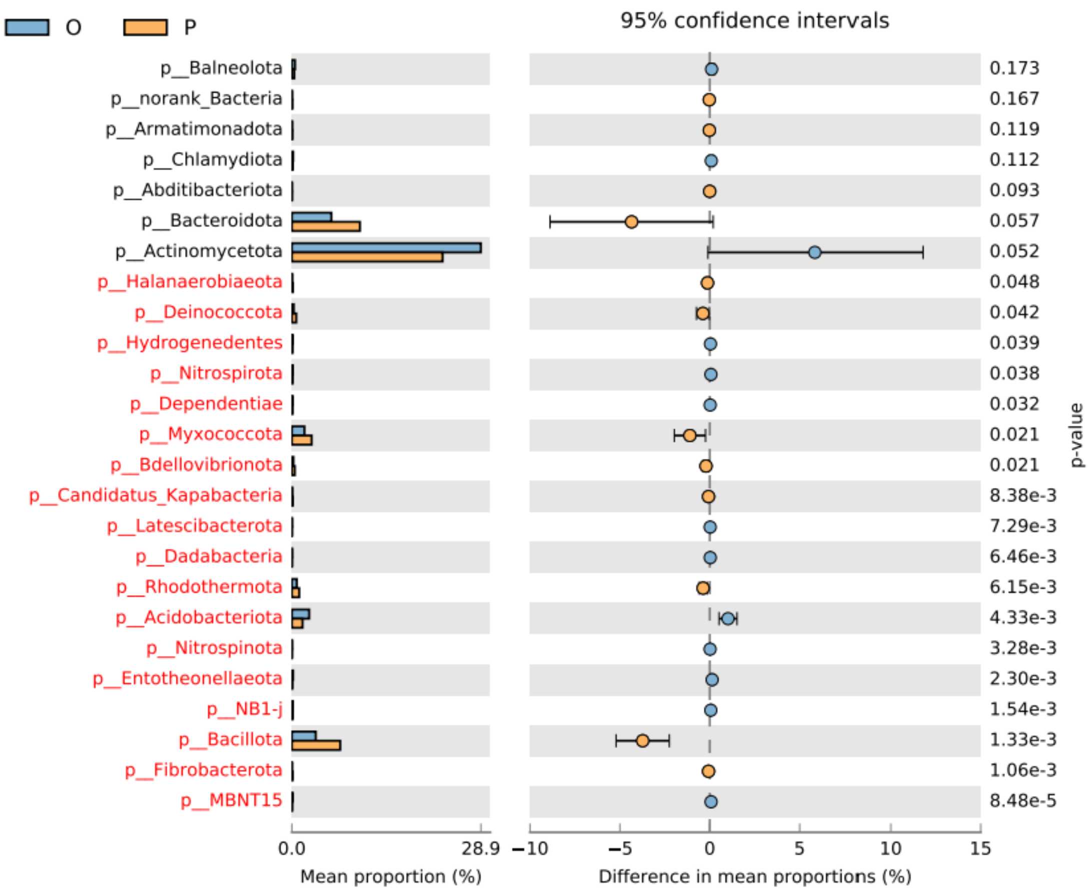
O
P
95% confidence intervals
p__Balneolota
p__norank_Bacteria
p__Armatimonadota
p__Chlamydiota
p__Abditibacteriota
p__Bacteroidota
p__Actinomycetota
p__Halanaerobiaeota
p__Deinococcota
p__Hydrogenedentes
p__Nitrospirota
p__Dependentiae
p__Myxococcota
p__Bdellovibrionota
p__Candidatus_Kapabacteria
p__Latescibacterota
p__Dadabacteria
p__Rhodothermota
p__Acidobacteriota
p__Nitrospinota
p__Entotheonellaeota
p__NB1-j
p__Bacillota
p__Fibrobacterota
p__MBNT15
0.173
0.167
0.119
0.112
0.093
0.057
0.052
0.048
0.042
0.039
0.038
0.032
0.021
0.021
8.38e-3
7.29e-3
6.46e-3
6.15e-3
4.33e-3
3.28e-3
2.30e-3
1.54e-3
1.33e-3
1.06e-3
8.48e-5
p-value
0.0
28.9
Mean proportion (%)
−10
−5
0
5
10
15
Difference in mean proportions (%)

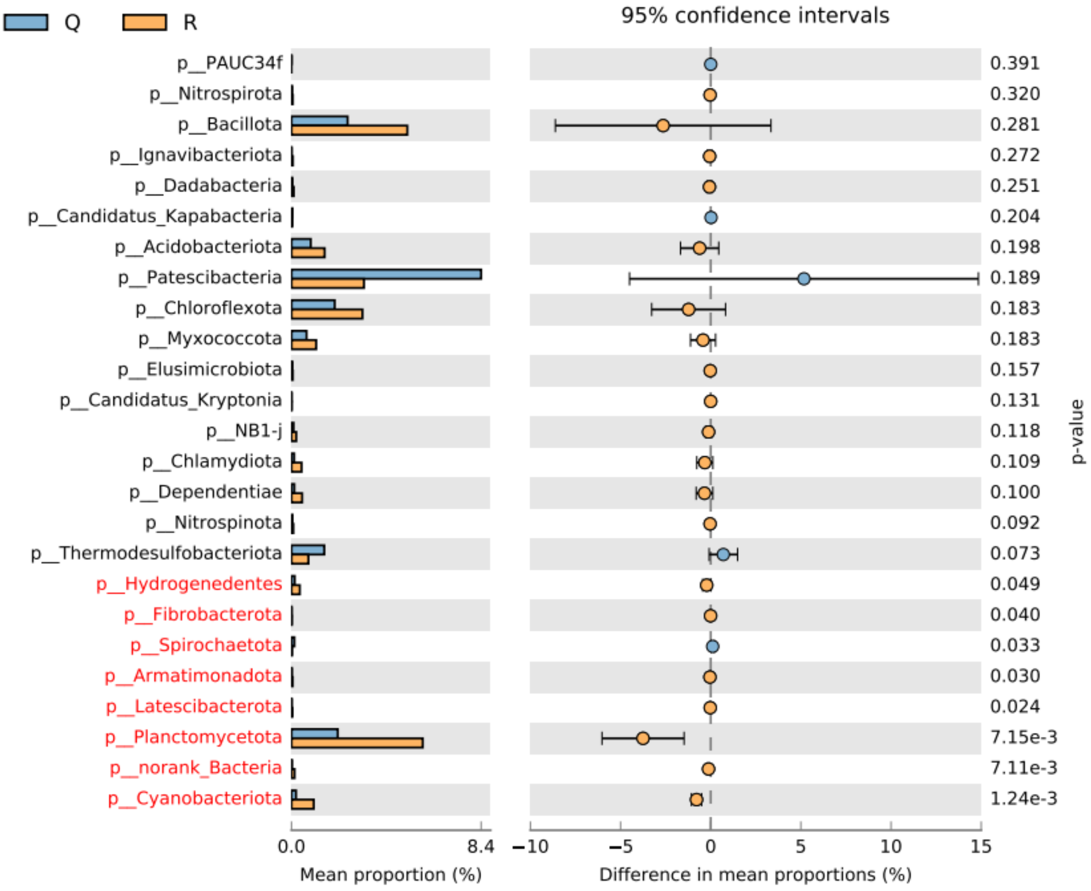
Q
R
95% confidence intervals
p__PAUC34f
p__Nitrospirota
p__Bacillota
p__Ignavibacteriota
p__Dadabacteria
p__Candidatus_Kapabacteria
p__Acidobacteriota
p__Patescibacteria
p__Chloroflexota
p__Myxococcota
p__Elusimicrobiota
p__Candidatus_Kryptonia
p__NB1-j
p__Chlamydiota
p__Dependentiae
p__Nitrospinota
p__Thermodesulfobacteriota
p__Hydrogenedentes
p__Fibrobacterota
p__Spirochaetota
p__Armatimonadota
p__Latescibacterota
p__Planctomycetota
p__norank_Bacteria
p__Cyanobacteriota
0.391
0.320
0.281
0.272
0.251
0.204
0.198
0.189
0.183
0.183
0.157
0.131
0.118
0.109
0.100
0.092
0.073
0.049
0.040
0.033
0.030
0.024
7.15e-3
7.11e-3
1.24e-3
p-value
0.0
8.4
Mean proportion (%)
−10
−5
0
5
10
15
Difference in mean proportions (%)

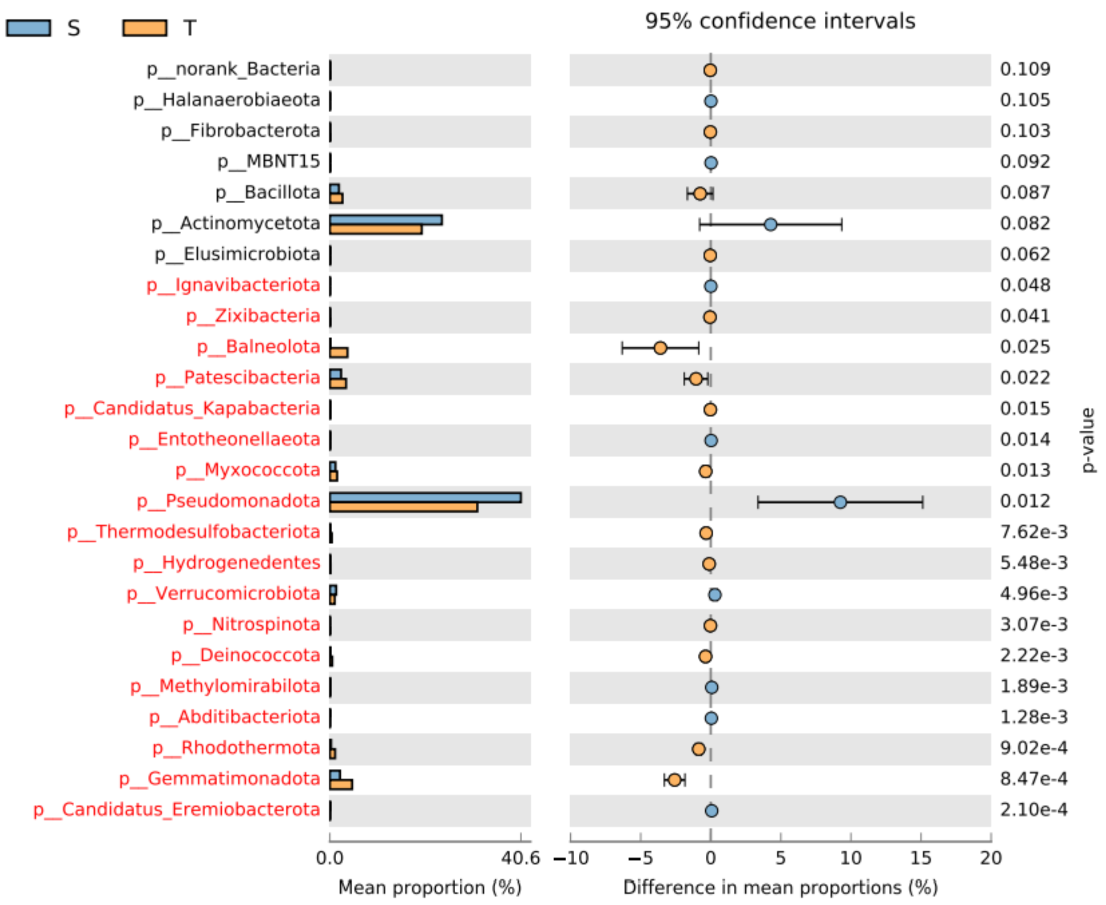

Part 2: Differential Analysis Results of Fungal Communities

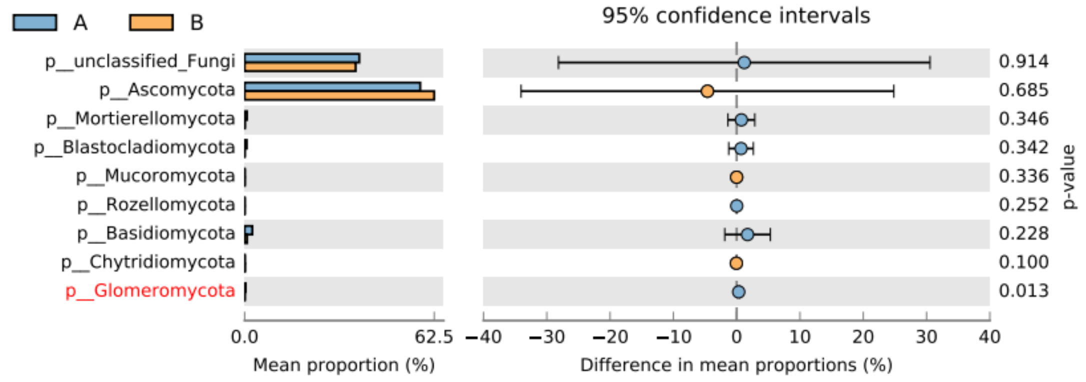

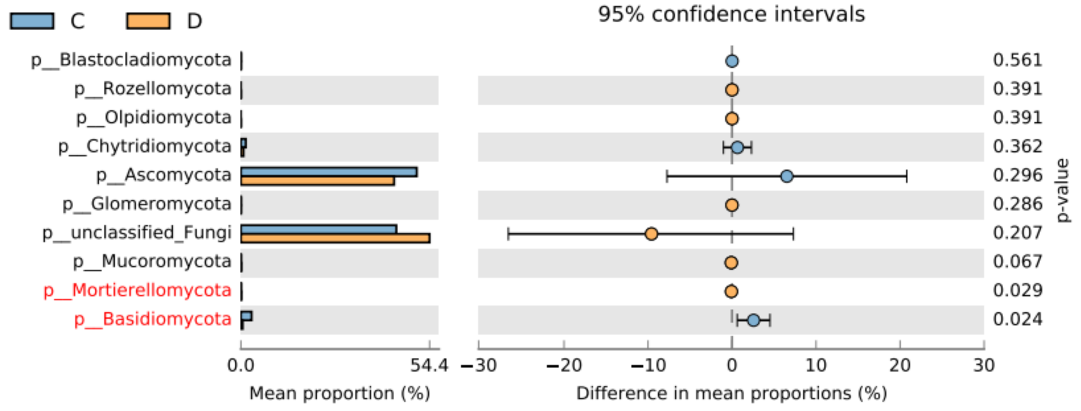

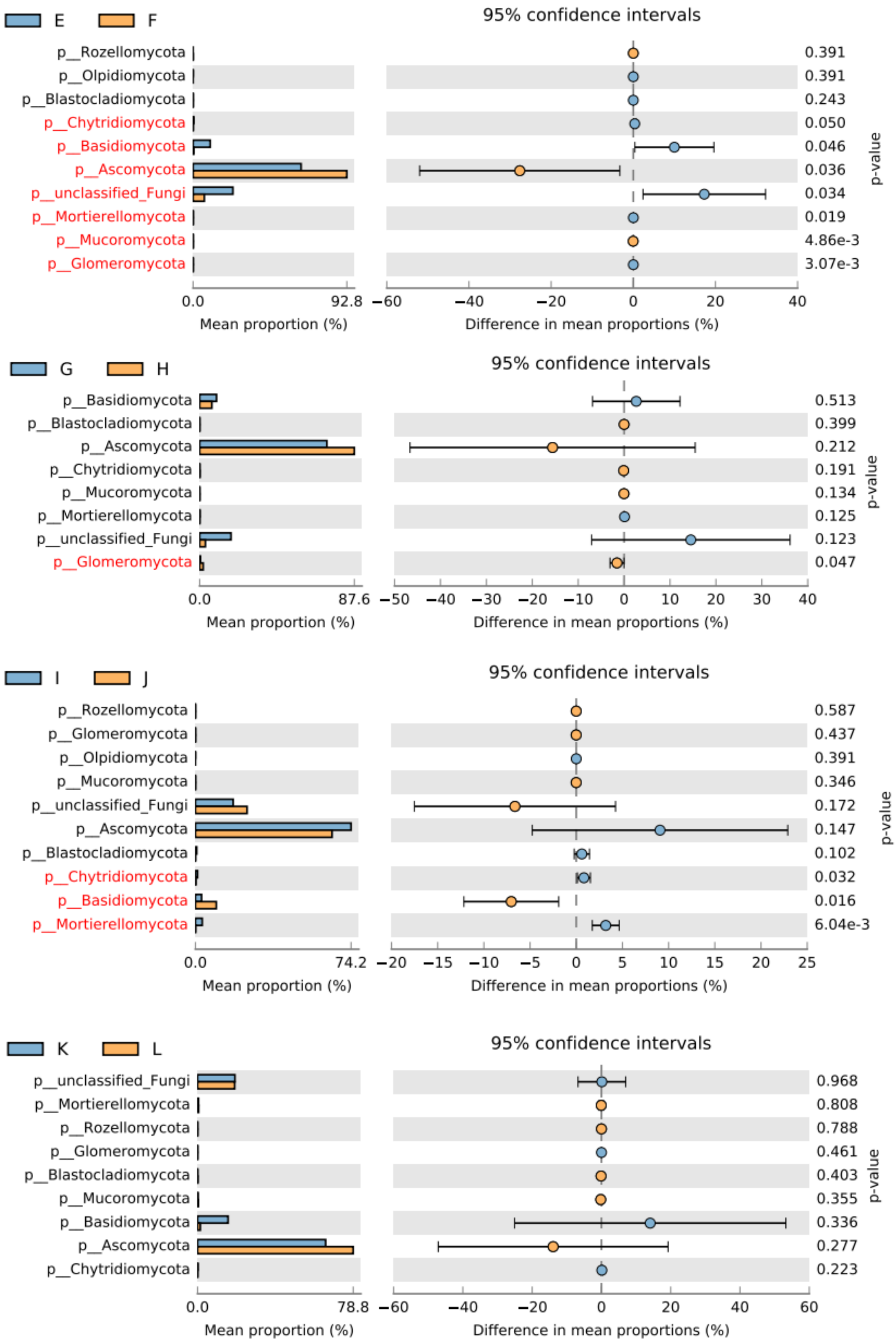

E
F
95% confidence intervals
p__Rozellomycota
p__Olpidiomycota
p__Blastocladiomycota
p__Chytridiomycota
p__Basidiomycota
p__Ascomycota
p__unclassified_Fungi
p__Mortierellomycota
p__Mucoromycota
p__Glomeromycota
0.391
0.391
0.243
0.050
0.046
0.036
0.034
0.019
4.86e-3
3.07e-3
p-value
0.0
92.8
Mean proportion (%)
−60
−40
−20
0
20
40
Difference in mean proportions (%)
G
H
95% confidence intervals
p__Basidiomycota
p__Blastocladiomycota
p__Ascomycota
p__Chytridiomycota
p__Mucoromycota
p__Mortierellomycota
p__unclassified_Fungi
p__Glomeromycota
0.513
0.399
0.212
0.191
0.134
0.125
0.123
0.047
p-value
0.0
87.6
Mean proportion (%)
−50
−40
−30
−20
−10
0
10
20
30
40
Difference in mean proportions (%)
I
J
95% confidence intervals
p__Rozellomycota
p__Glomeromycota
p__Olpidiomycota
p__Mucoromycota
p__unclassified_Fungi
p__Ascomycota
p__Blastocladiomycota
p__Chytridiomycota
p__Basidiomycota
p__Mortierellomycota
0.587
0.437
0.391
0.346
0.172
0.147
0.102
0.032
0.016
6.04e-3
p-value
0.0
74.2
Mean proportion (%)
−20
−15
−10
−5
0
5
10
15
20
25
Difference in mean proportions (%)
K
L
95% confidence intervals
p__unclassified_Fungi
p__Mortierellomycota
p__Rozellomycota
p__Glomeromycota
p__Blastocladiomycota
p__Mucoromycota
p__Basidiomycota
p__Ascomycota
p__Chytridiomycota
0.968
0.808
0.788
0.461
0.403
0.355
0.336
0.277
0.223
p-value
0.0
78.8
Mean proportion (%)
−60
−40
−20
0
20
40
60
Difference in mean proportions (%)

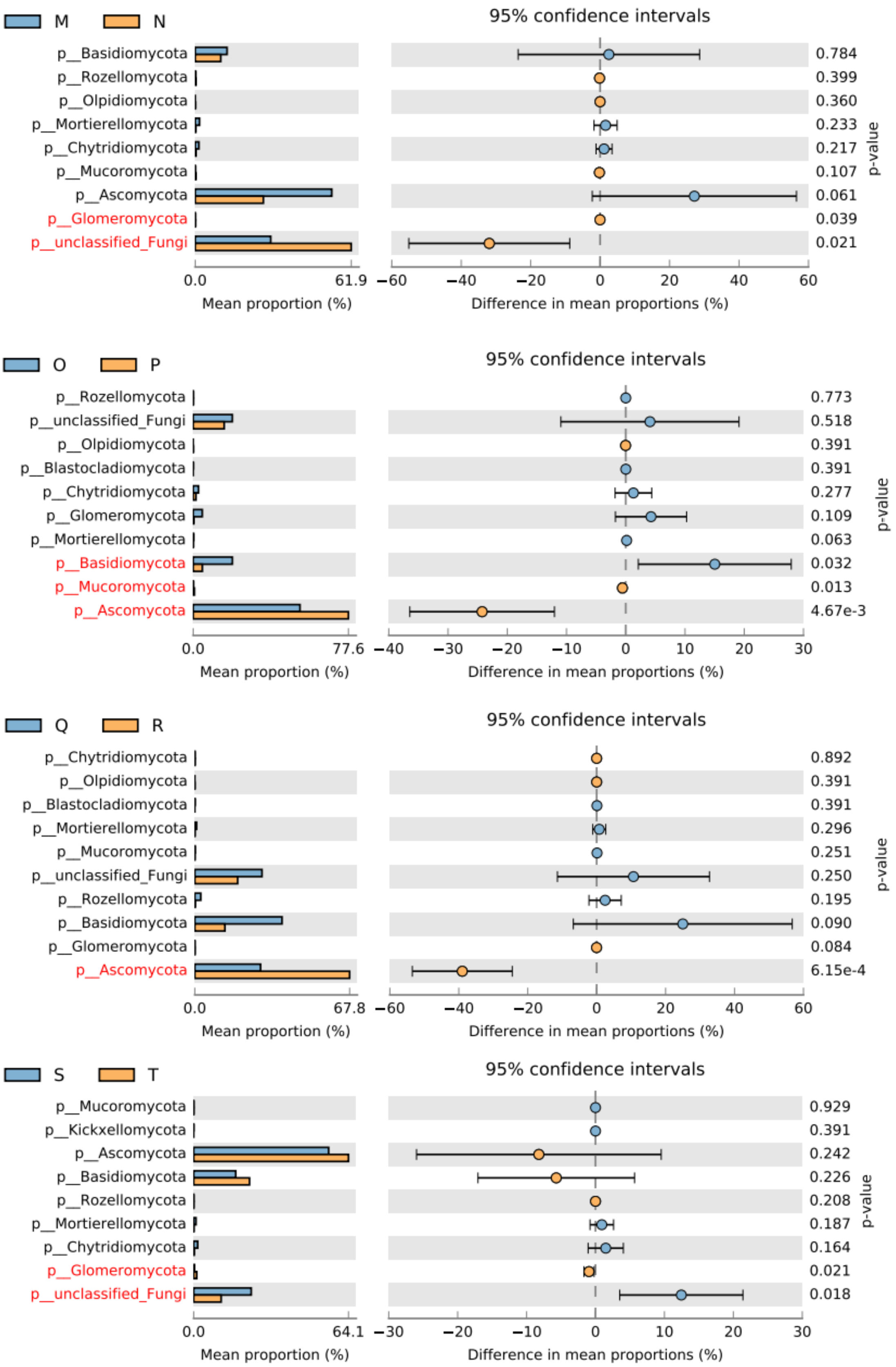

M
N
95% confidence intervals
p__Basidiomycota
p__Rozellomycota
p__Olpidiomycota
p__Mortierellomycota
p__Chytridiomycota
p__Mucoromycota
p__Ascomycota
p__Glomeromycota
p__unclassified_Fungi
0.784
0.399
0.360
0.233
0.217
0.107
0.061
0.039
0.021
p-value
0.0
61.9
−60
−40
−20
0
20
40
60
Mean proportion (%)
Difference in mean proportions (%)
O
P
95% confidence intervals
p__Rozellomycota
p__unclassified_Fungi
p__Olpidiomycota
p__Blastocladiomycota
p__Chytridiomycota
p__Glomeromycota
p__Mortierellomycota
p__Basidiomycota
p__Mucoromycota
p__Ascomycota
0.773
0.518
0.391
0.391
0.277
0.109
0.063
0.032
0.013
4.67e-3
p-value
0.0
77.6
−40
−30
−20
−10
0
10
20
30
Mean proportion (%)
Difference in mean proportions (%)
Q
R
95% confidence intervals
p__Chytridiomycota
p__Olpidiomycota
p__Blastocladiomycota
p__Mortierellomycota
p__Mucoromycota
p__unclassified_Fungi
p__Rozellomycota
p__Basidiomycota
p__Glomeromycota
p__Ascomycota
0.892
0.391
0.391
0.296
0.251
0.250
0.195
0.090
0.084
6.15e-4
p-value
0.0
67.8
−60
−40
−20
0
20
40
60
Mean proportion (%)
Difference in mean proportions (%)
S
T
95% confidence intervals
p__Mucoromycota
p__Kickxellomycota
p__Ascomycota
p__Basidiomycota
p__Rozellomycota
p__Mortierellomycota
p__Chytridiomycota
p__Glomeromycota
p__unclassified_Fungi
0.929
0.391
0.242
0.226
0.208
0.187
0.164
0.021
0.018
p-value
0.0
64.1
−30
−20
−10
0
10
20
30
Mean proportion (%)
Difference in mean proportions (%)
