# Supplementary material for: Rhizosphere Ion Composition Shapes Microbial Communities and Is Associated with Plant Growth Variation in Saline–Alkali Soils
Source: Microorganisms. 2026 Jun 14;14(6):1333. doi: 10.3390/microorganisms14061333 (PMC13305541; doi:10.3390/microorganisms14061333)
Supplement: Supplementary file 1 [file microorganisms-14-01333-s001.zip › Supplementary File S3.pdf]

Supplementary File S3. DESeq2 differential analysis results

Part 1: Differential Analysis Results of Bacterial Communities

A vs B:

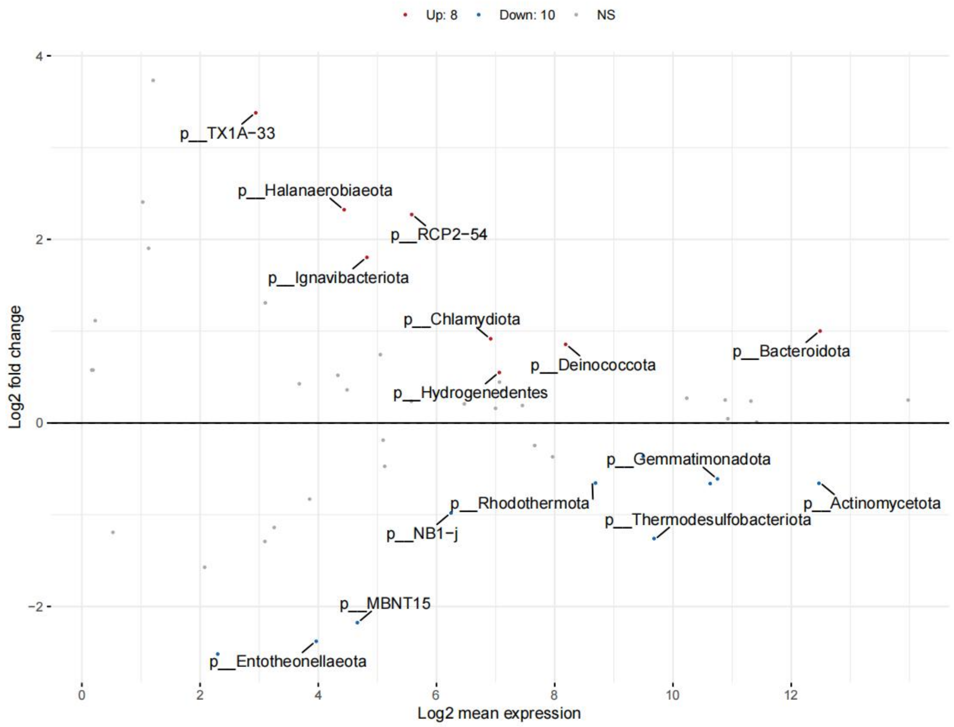

C vs D:

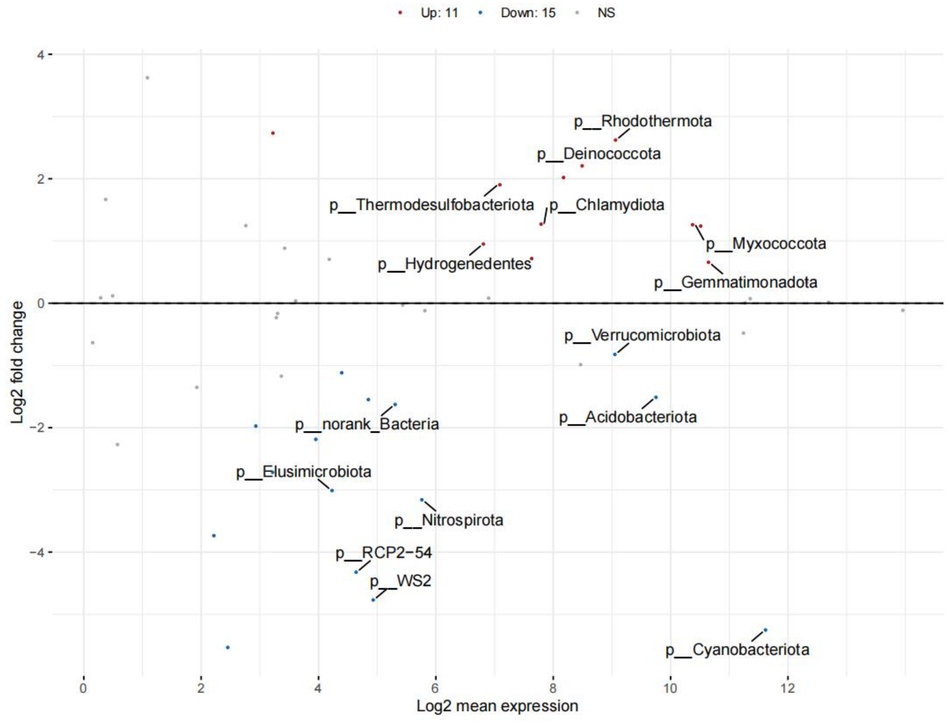

E vs F:

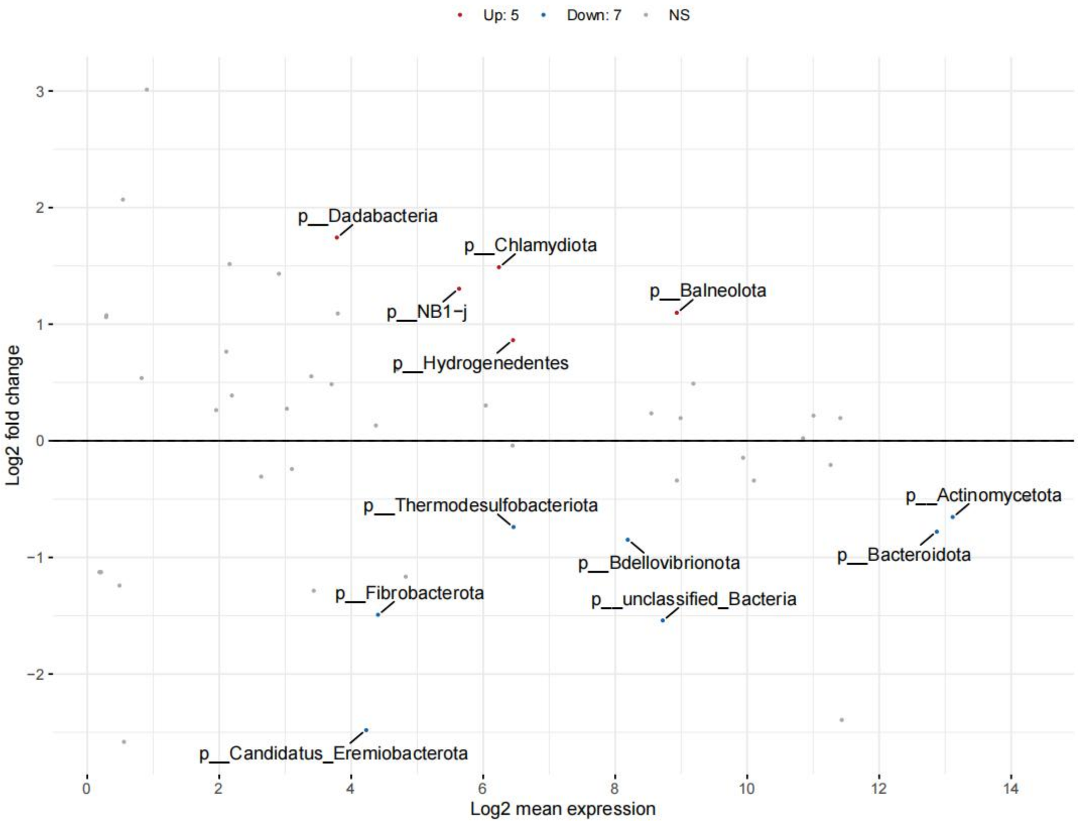
Up: 5
Down: 7
NS
p__Dadabacteria
p__Chlamydiota
p__NB1−j
p__Balneolota
p__Hydrogenedentes
p__Thermodesulfobacteriota
p__Actinomycetota
p__Bacteroidota
p__Bdellovibrionota
p__Fibrobacterota
p__unclassified_Bacteria
p__Candidatus_Eremiobacterota
Log2 fold change
Log2 mean expression

G vs H:

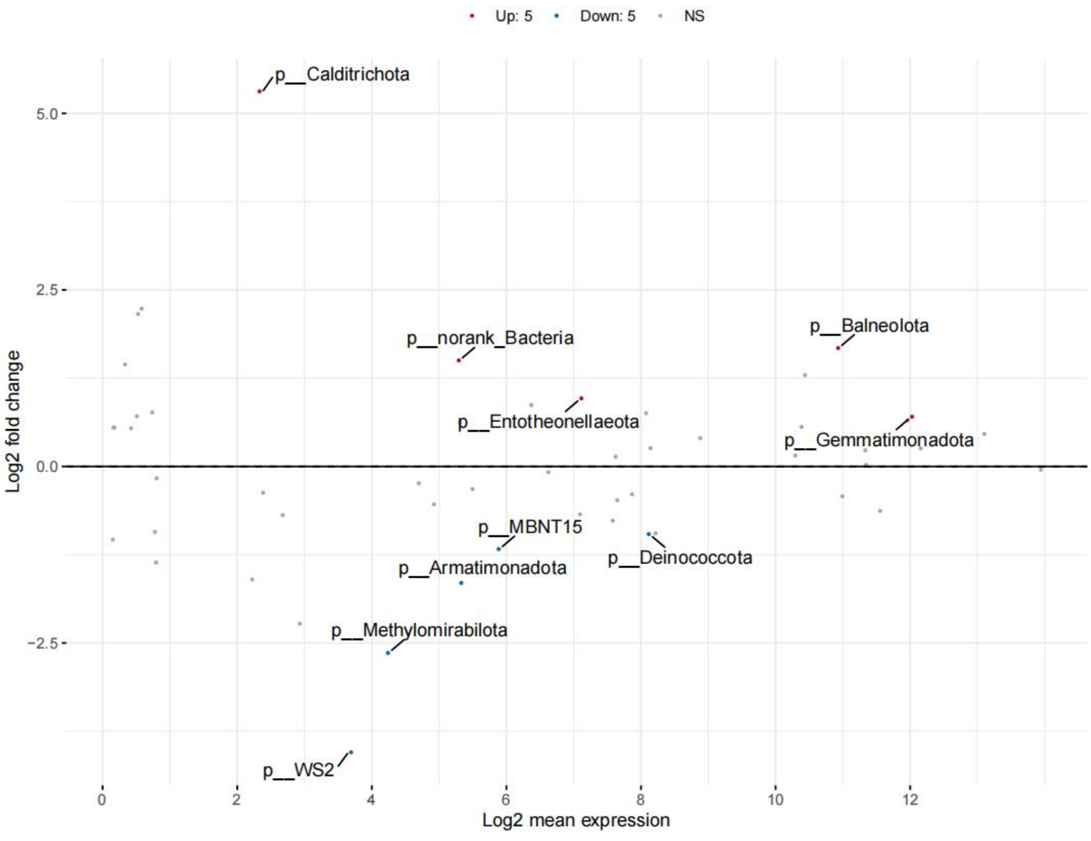
Up: 5
Down: 5
NS
p__Calditrichota
p__norank_Bacteria
p__Balneolota
p__Entotheonellaeota
p__Gemmatimonadota
p__MBNT15
p__Deinococcota
p__Armatimonadota
p__Methylomirabilota
p__WS2
Log2 fold change
Log2 mean expression

I vs J:

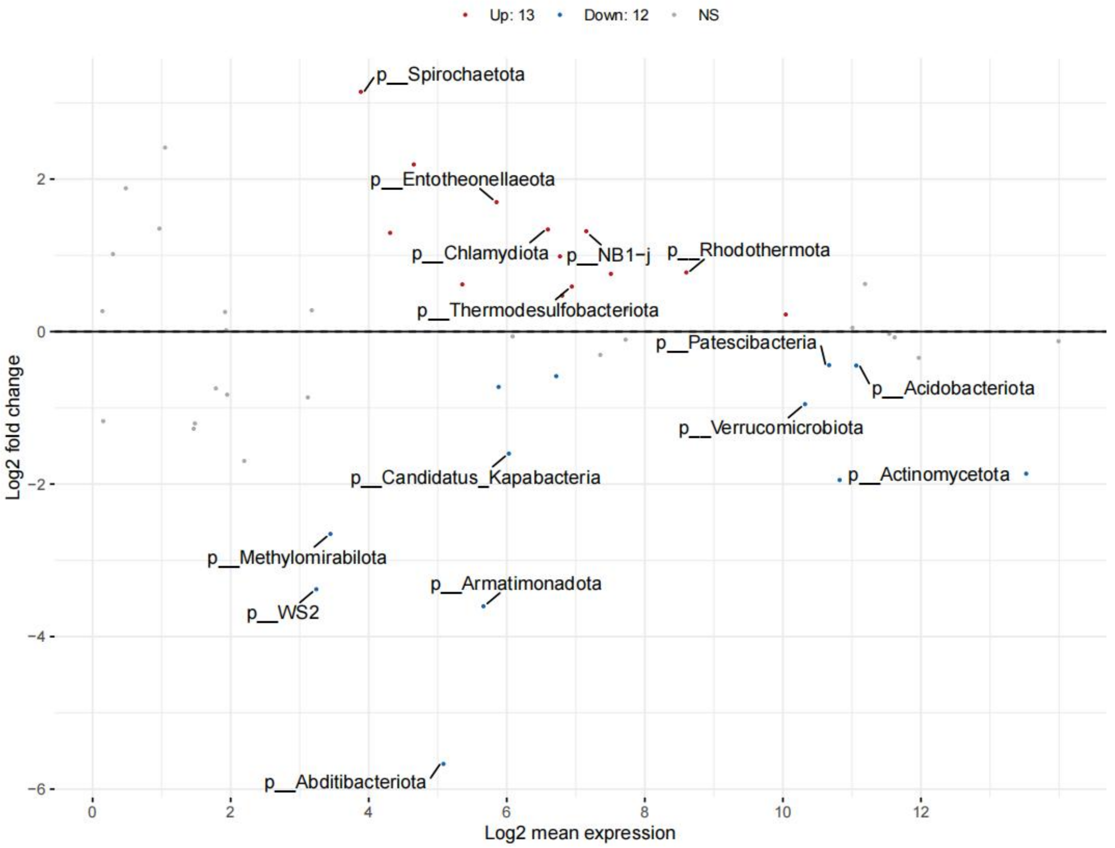
Up: 13
Down: 12
NS
p__Spirochaetota
p__Entotheonellaeota
p__Chlamydiota
p__NB1−j
p__Rhodothermota
p__Thermodesulfobacteriota
p__Patescibacteria
p__Acidobacteriota
p__Verrucomicrobiota
p__Candidatus_Kapabacteria
p__Actinomycetota
p__Methylomirabilota
p__Armatimonadota
p__WS2
p__Abditibacteriota
Log2 fold change
Log2 mean expression
2
0
−2
−4
−6
0
2
4
6
8
10
12

K vs L:

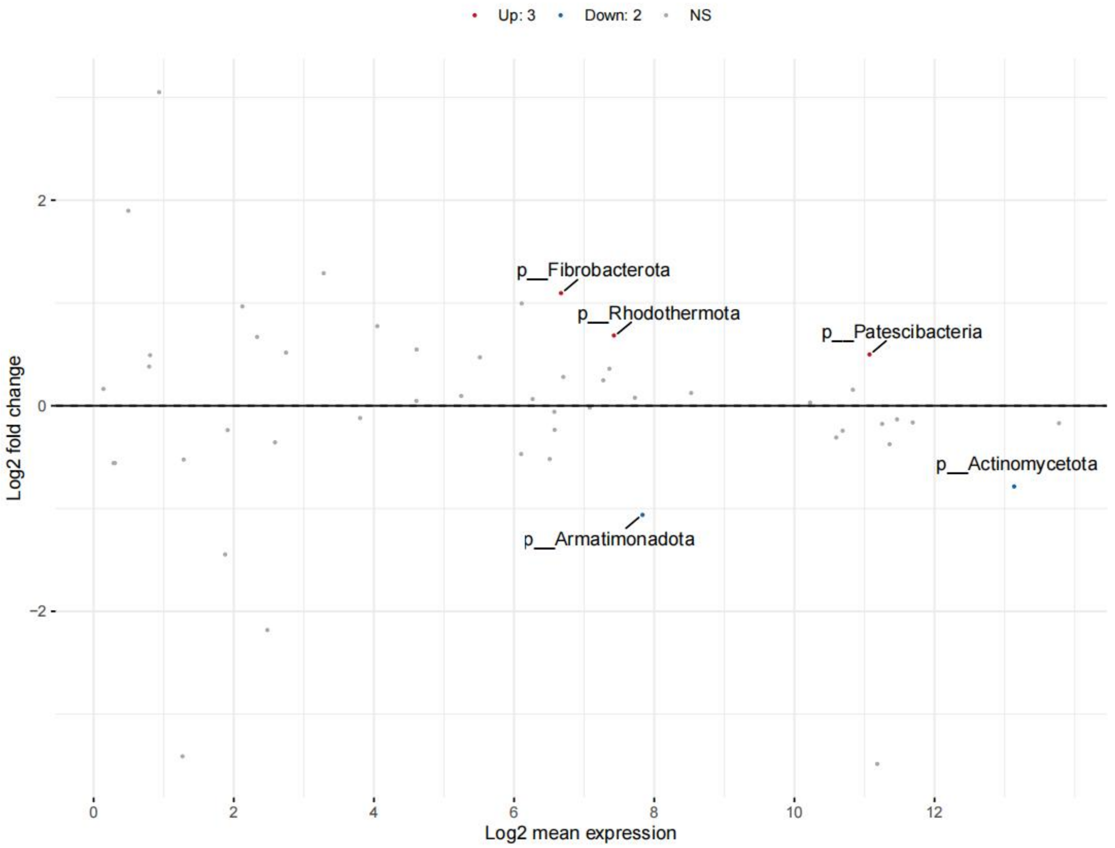
Up: 3
Down: 2
NS
p__Fibrobacterota
p__Rhodothermota
p__Patescibacteria
p__Actinomycetota
p__Armatimonadota
Log2 fold change
Log2 mean expression
2
0
−2
0
2
4
6
8
10
12

M vs N:

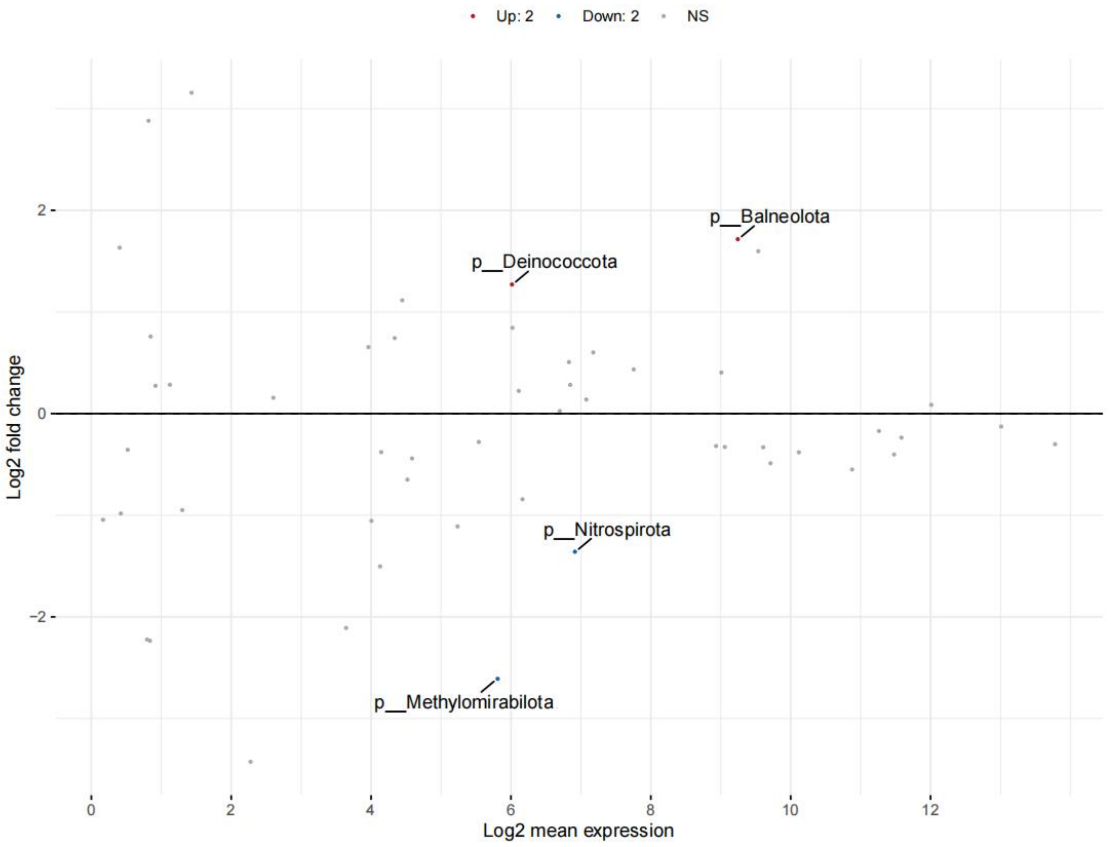
Up: 2
Down: 2
NS
p__Balneolota
p__Deinococcota
p__Nitrospirota
p__Methylomirabilota
Log2 fold change
Log2 mean expression
2
0
-2
0
2
4
6
8
10
12

O vs P:

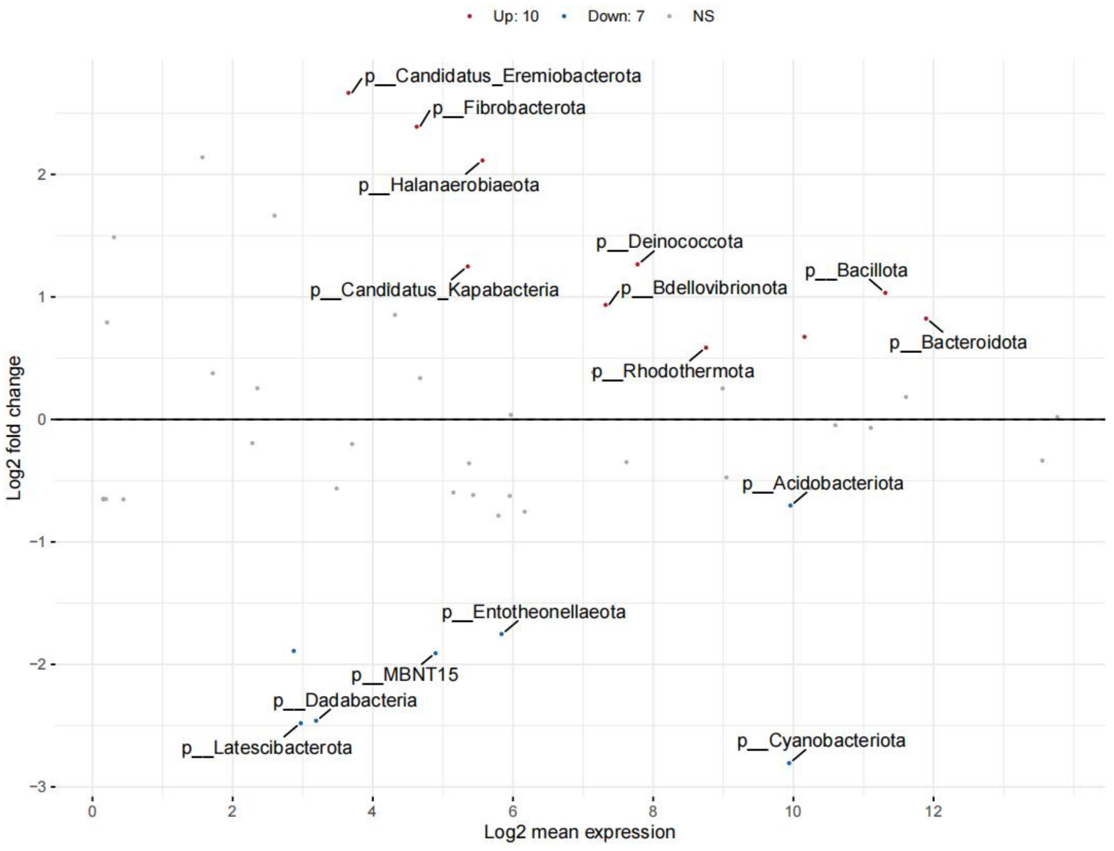
Up: 10
Down: 7
NS
p__Candidatus_Eremiobacterota
p__Fibrobacterota
p__Halanaerobiaeota
p__Deinococcota
p__Bacillota
p__Candidatus_Kapabacteria
p__Bdellovibrionota
p__Bacteroidota
p__Rhodothermota
p__Acidobacteriota
p__Entotheonellaeota
p__MBNT15
p__Dadabacteria
p__Latescibacterota
p__Cyanobacteriota
Log2 fold change
Log2 mean expression
2
1
0
-1
-2
-3
0
2
4
6
8
10
12

Q vs R:

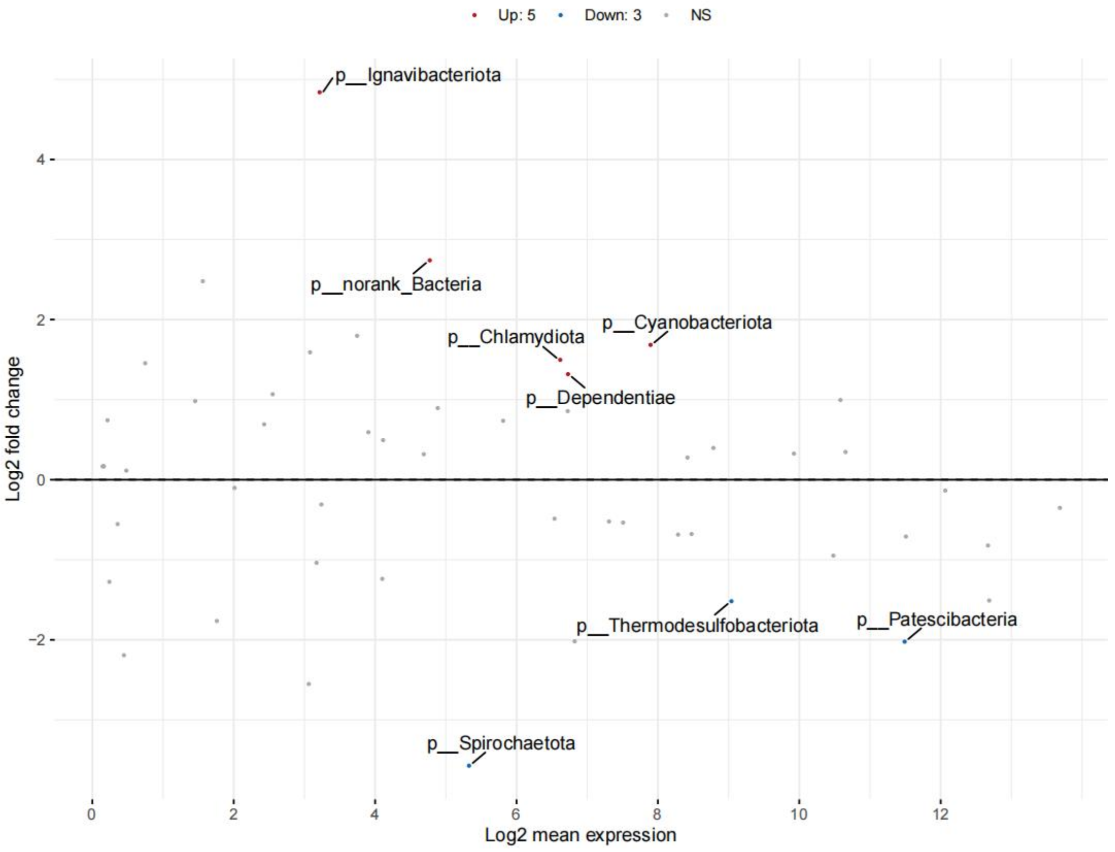

Up: 5
Down: 3
NS
p__Ignavibacteriota
p__norank_Bacteria
p__Cyanobacteriota
p__Chlamydiota
p__Dependentiae
p__Thermodesulfobacteriota
p__Patescibacteria
p__Spirochaetota
Log2 fold change
Log2 mean expression
4
2
0
-2
0
2
4
6
8
10
12

S vs T:

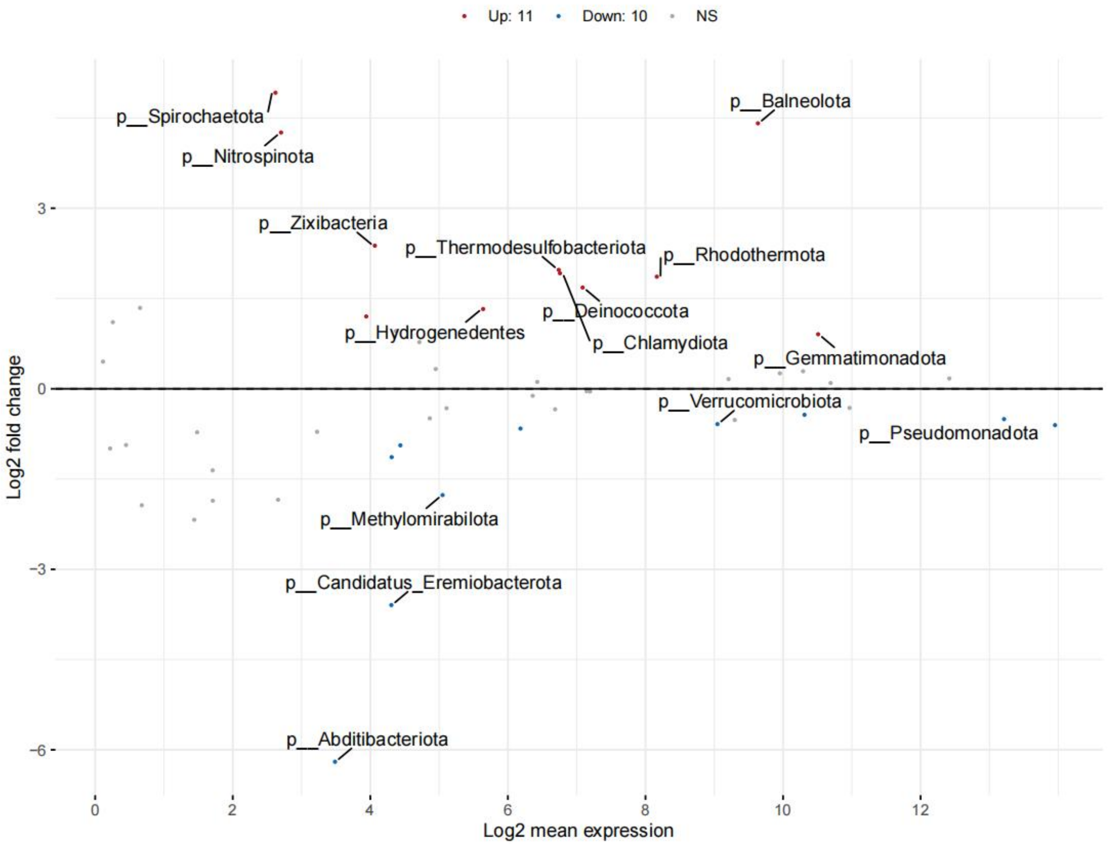

Up: 11
Down: 10
NS
p__Spirochaetota
p__Nitrospinota
p__Balneolota
p__Zixibacteria
p__Thermodesulfobacteriota
p__Rhodothermota
p__Deinococcota
p__Hydrogenedentes
p__Chlamydiota
p__Gemmatimonadota
p__Verrucomicrobiota
p__Pseudomonadota
p__Methylomirabilota
p__Candidatus_Eremiobacterota
p__Abditibacteriota
Log2 fold change
Log2 mean expression
3
0
-3
-6
0
2
4
6
8
10
12

Part 2: Differential Analysis Results of Fungal Communities

A vs B:

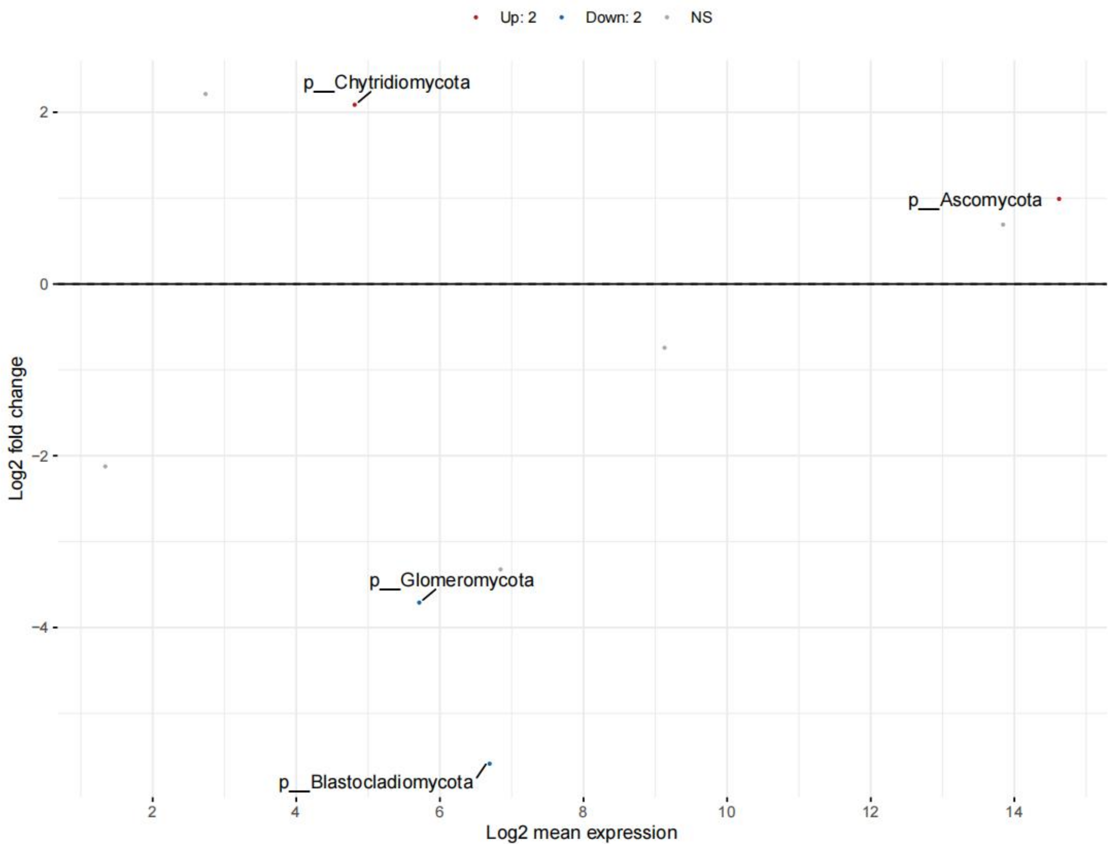

C vs D:

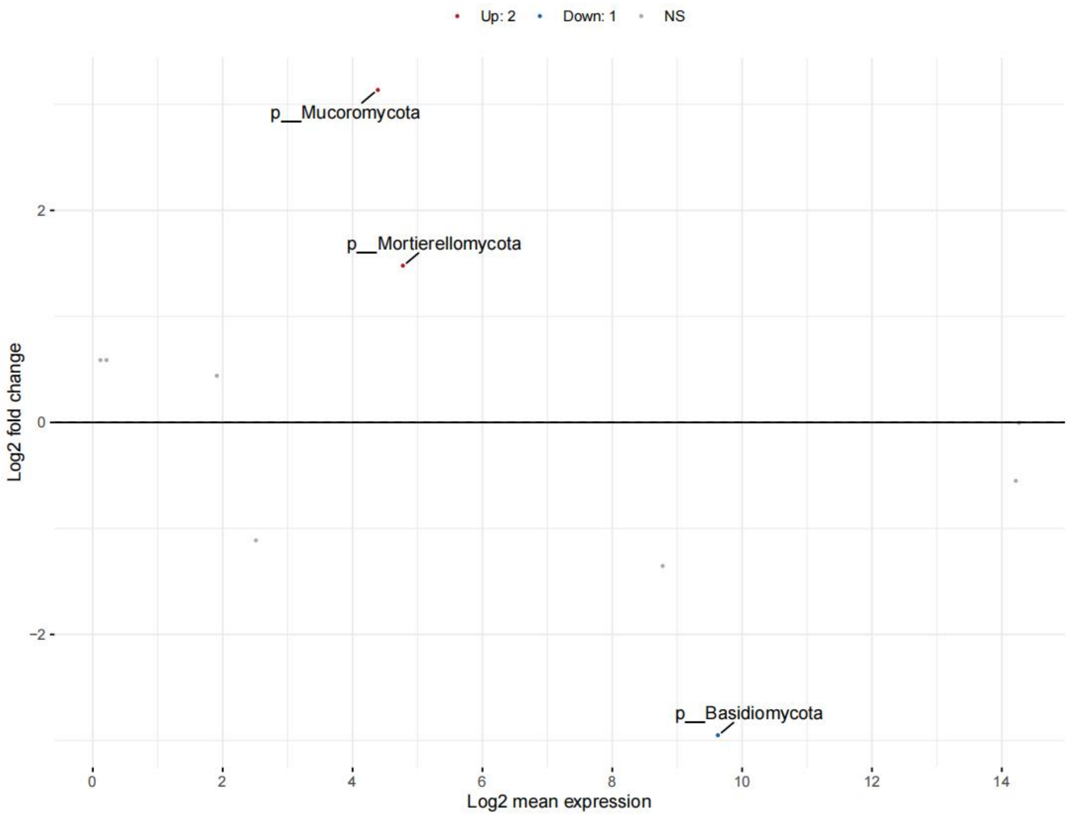

E vs F:

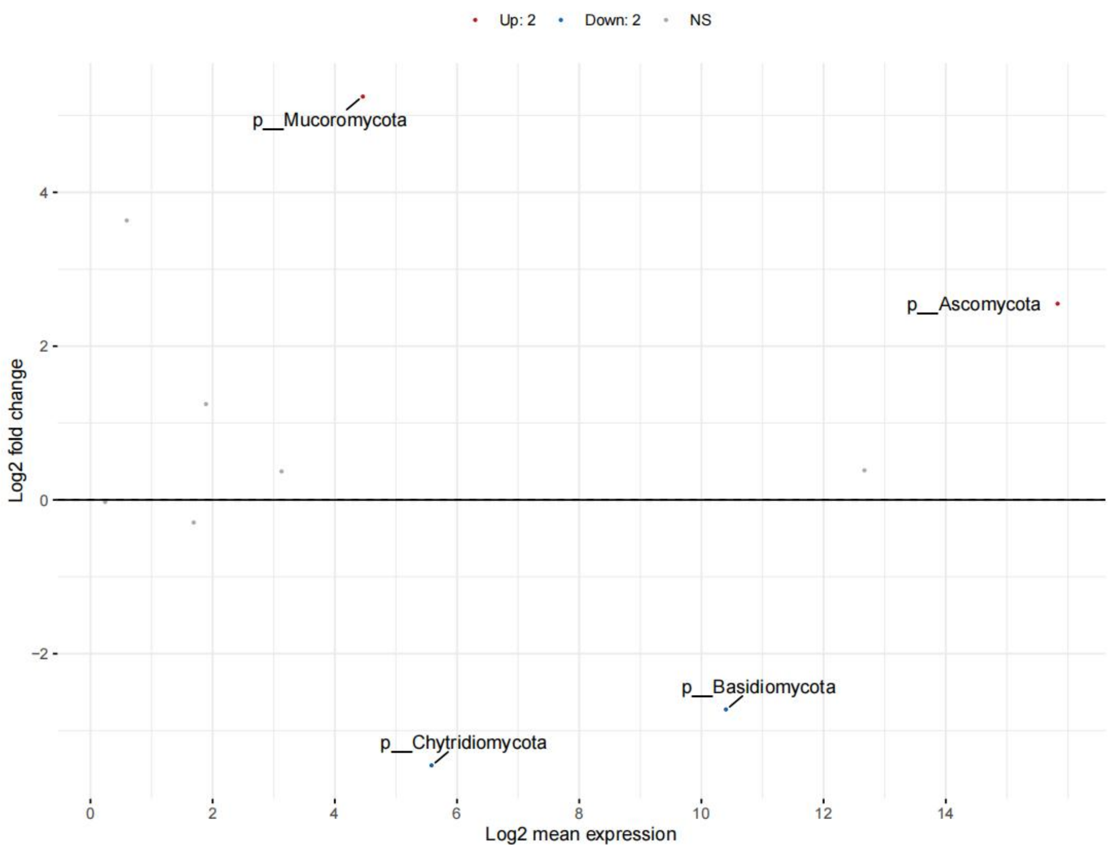
Up: 2
Down: 2
NS
p__Mucoromycota
p__Ascomycota
p__Basidiomycota
p__Chytridiomycota
Log2 fold change
Log2 mean expression

G vs H:

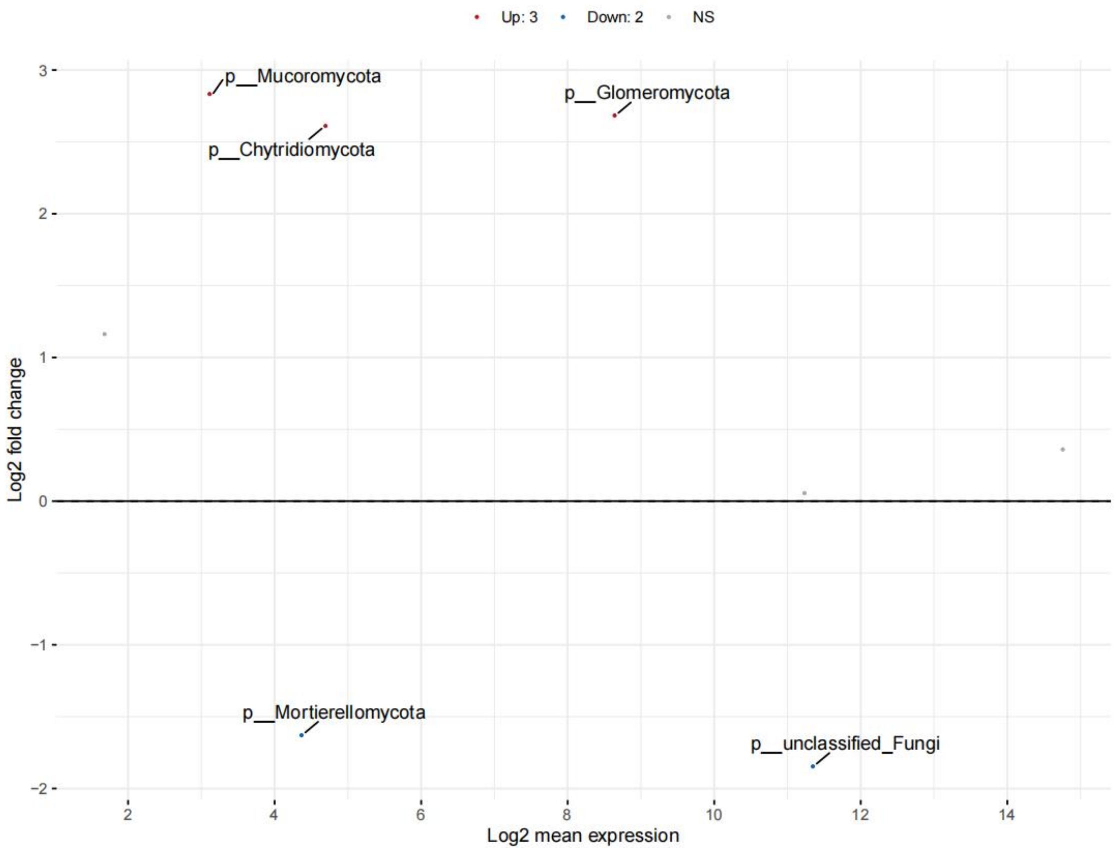
Up: 3
Down: 2
NS
p__Mucoromycota
p__Glomeromycota
p__Chytridiomycota
p__Mortierellomycota
p__unclassified_Fungi
Log2 fold change
Log2 mean expression

I vs J:

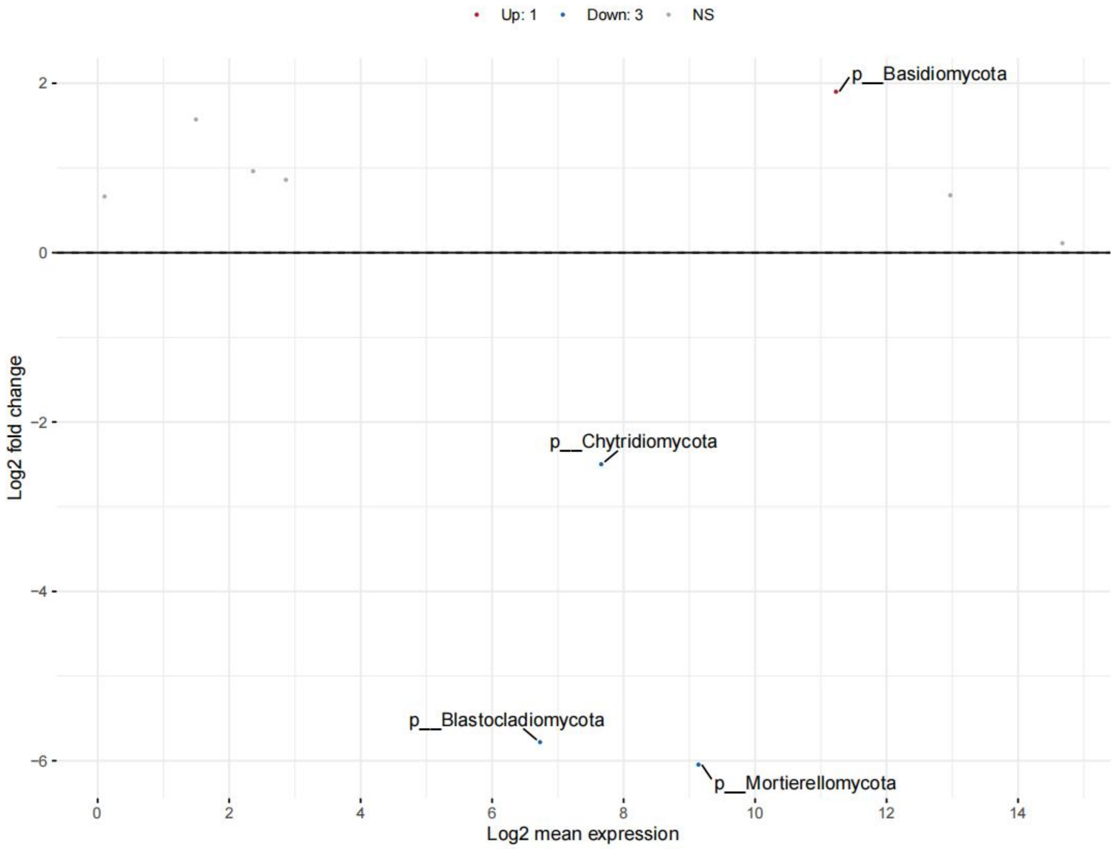
Up: 1
Down: 3
NS
p__Basidiomycota
p__Chytridiomycota
p__Blastocladiomycota
p__Mortierellomycota
Log2 fold change
Log2 mean expression
2
0
−2
−4
−6
0
2
4
6
8
10
12
14

K vs L:

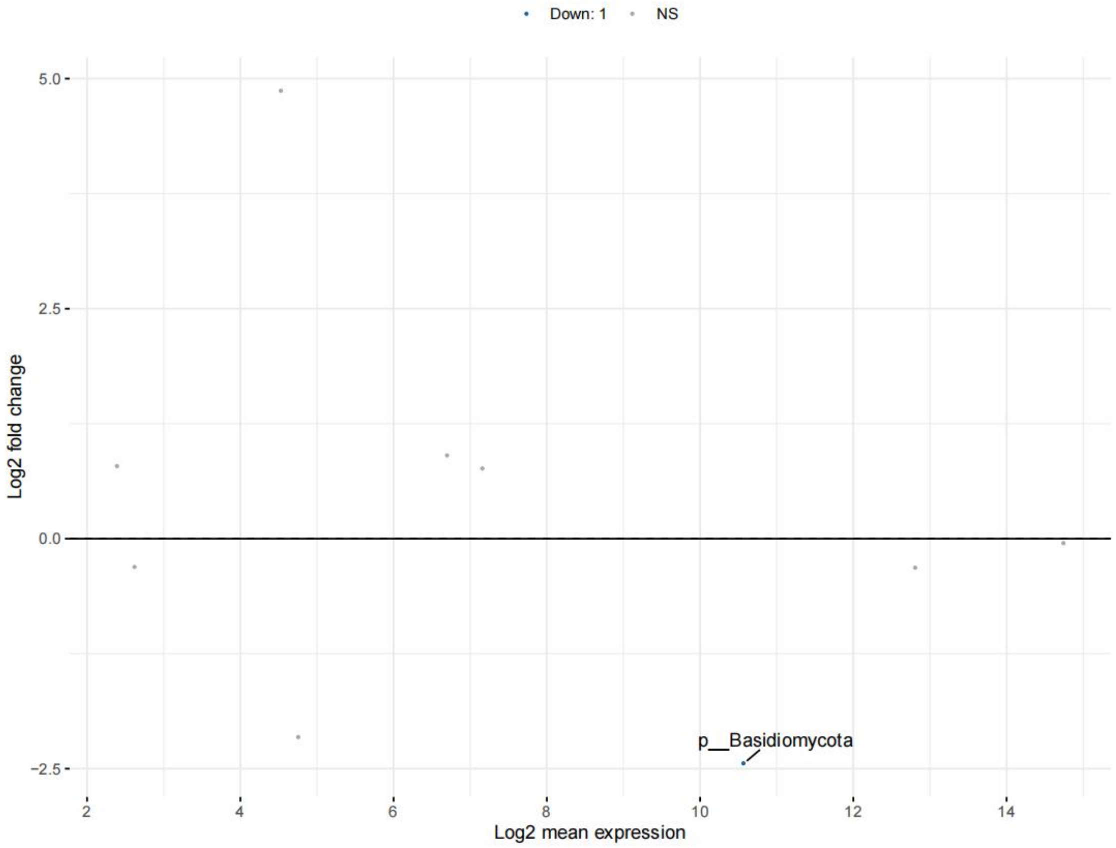
Down: 1
NS
p__Basidiomycota
Log2 fold change
Log2 mean expression
5.0
2.5
0.0
−2.5
2
4
6
8
10
12
14

M vs N:

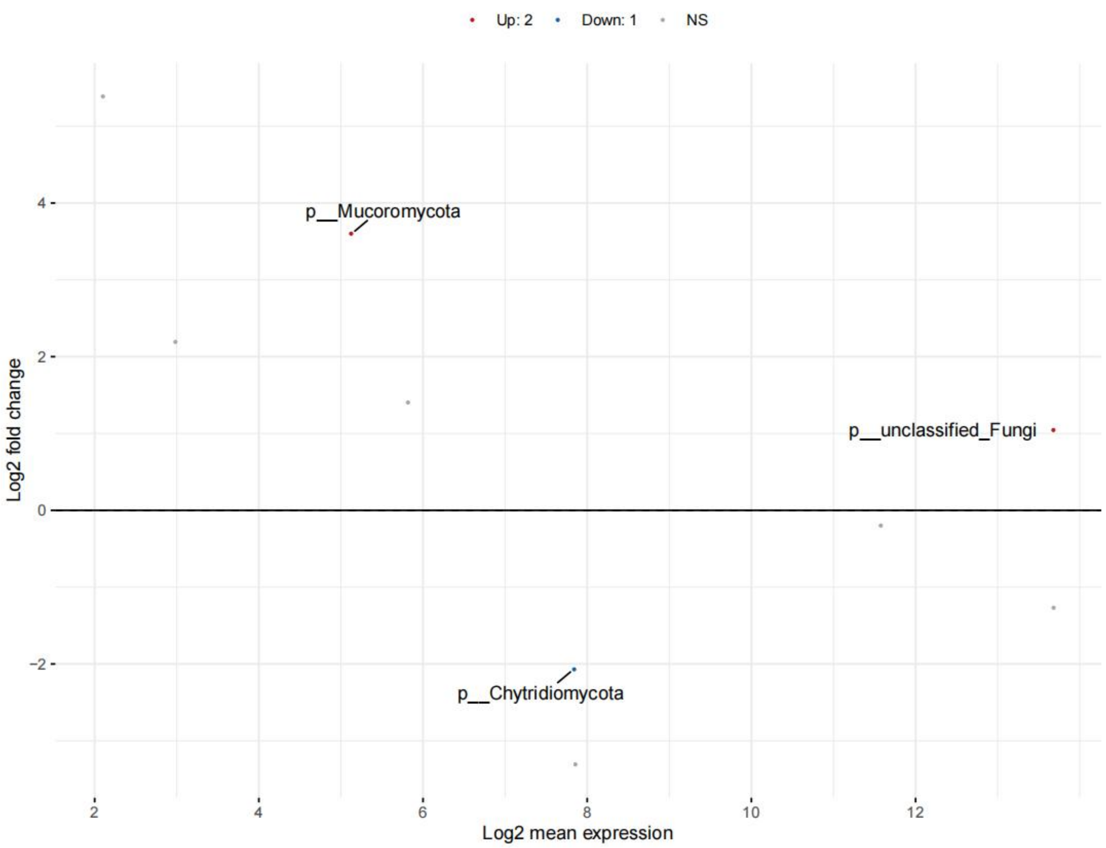

Up: 2
Down: 1
NS
p__Mucoromycota
p__unclassified_Fungi
p__Chytridiomycota
Log2 fold change
Log2 mean expression
4
2
0
−2
2
4
6
8
10
12

O vs P:

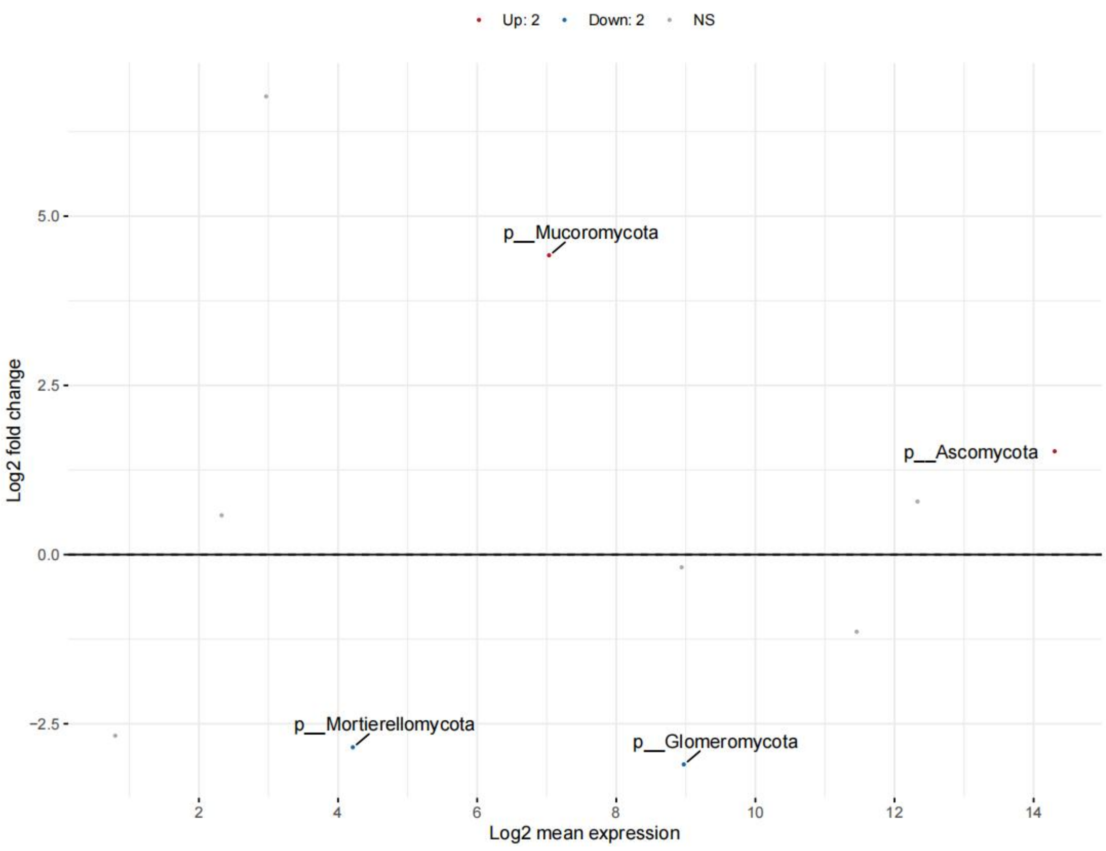

Up: 2
Down: 2
NS
p__Mucoromycota
p__Ascomycota
p__Mortierellomycota
p__Glomeromycota
Log2 fold change
Log2 mean expression
5.0
2.5
0.0
−2.5
2
4
6
8
10
12
14

Q vs R:

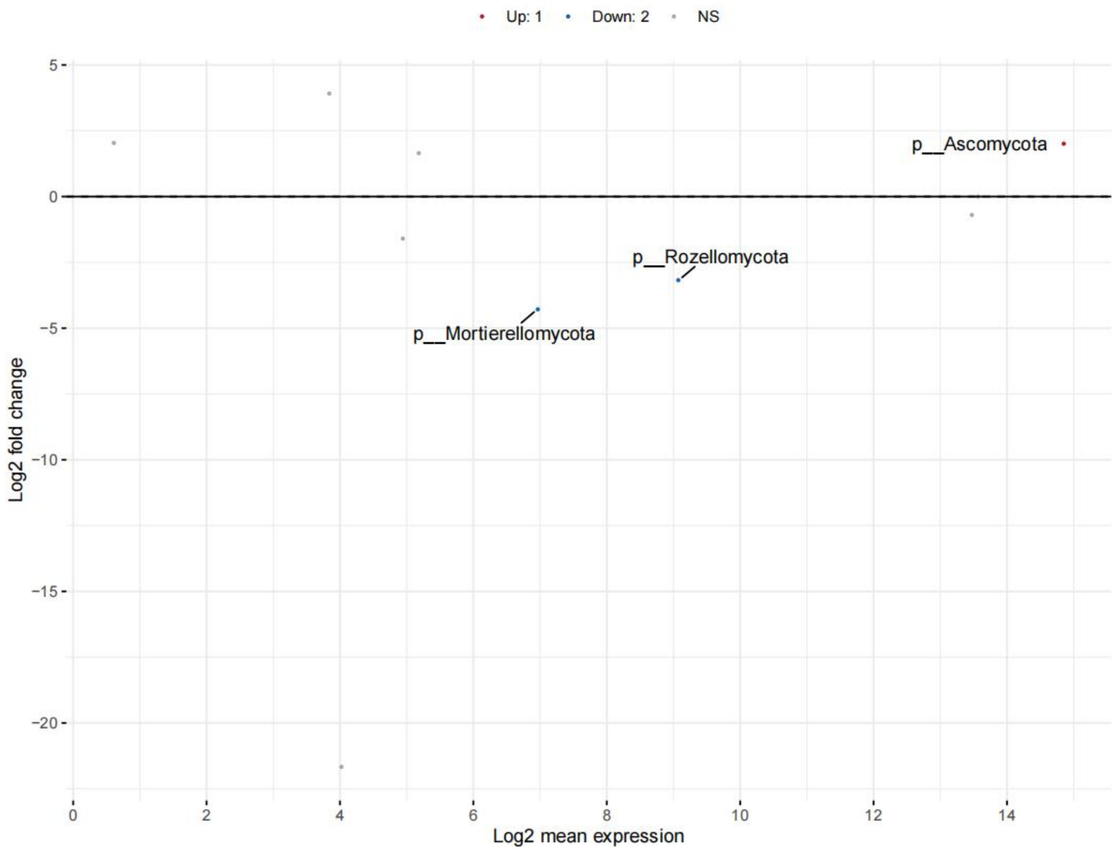

Up: 1
Down: 2
NS
p__Ascomycota
p__Rozellomycota
p__Mortierellomycota
Log2 fold change
Log2 mean expression

S vs T:

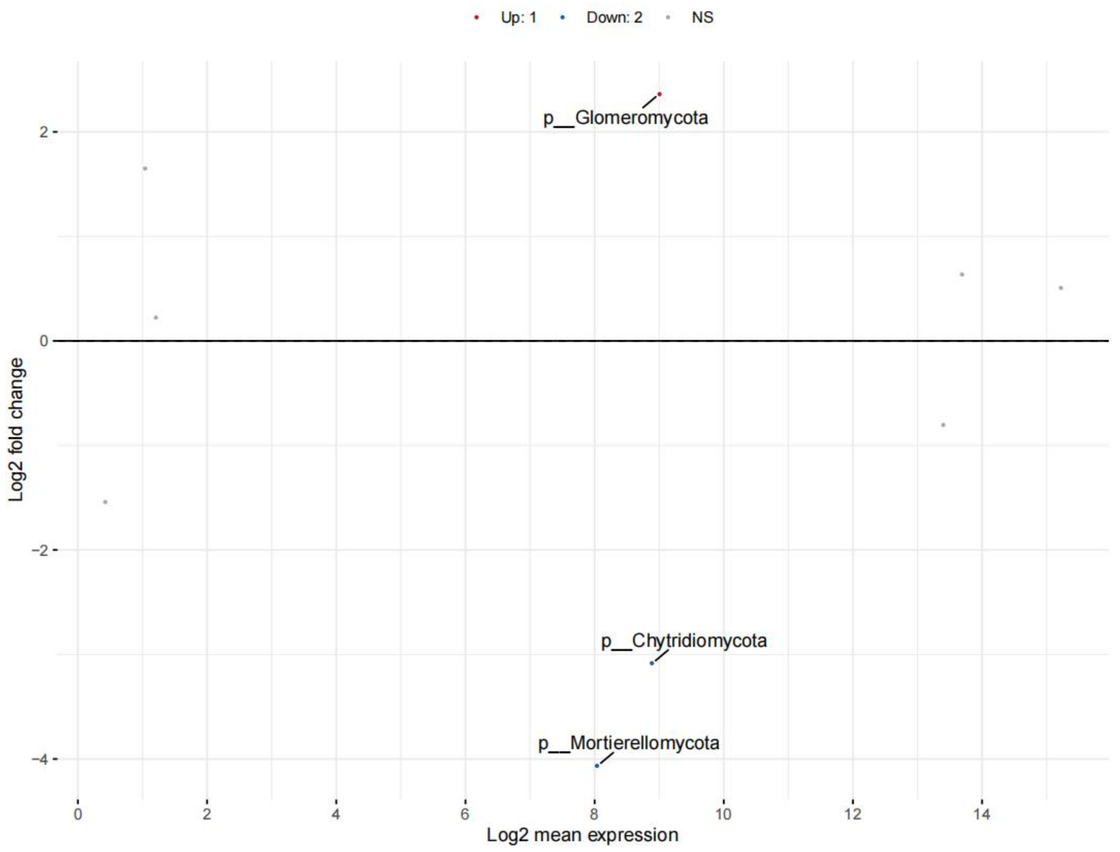

Up: 1
Down: 2
NS
p__Glomeromycota
p__Chytridiomycota
p__Mortierellomycota
Log2 fold change
Log2 mean expression
